# The role of different types of programmed cell death in myocardial fibrosis: from mechanisms to therapeutics

**DOI:** 10.3389/fcvm.2026.1748166

**Published:** 2026-04-08

**Authors:** Lingyun Wang, Guoci Lu, Lei Xiang, Yuzhen Fan, Kai Liu

**Affiliations:** 1 School of Traditional Chinese and Western Medicine, Gansu University of Chinese Medicine, Lanzhou, China; 2 Department of Clinical Medicine, Gansu Medical College, Pingliang, China

**Keywords:** cardiac fibroblasts, cardiovascular diseases, myocardial fibrosis, programmed cell death, therapeutic targets

## Abstract

Cardiovascular diseases remain one of the leading causes of death in both developed and developing countries, imposing a substantial disease burden. Myocardial fibrosis—one of the most common pathological changes in the development of cardiovascular diseases—is characterized by excessive deposition of the extracellular matrix (ECM). Myocardial fibrosis can lead to impaired cardiac systolic and diastolic functions. Programmed cell death (PCD) plays an important role in the occurrence and development of myocardial fibrosis. Different types of PCD—such as apoptosis, autophagic programmed cell death, ferroptosis, necroptosis, and pyroptosis—affect the activation and proliferation of cardiac fibroblasts, as well as the synthesis and degradation of ECM through their unique signaling pathways. An in-depth understanding of the mechanisms of programmed cell death in myocardial fibrosis is expected to provide new targets and strategies for the treatment of cardiovascular diseases. Therefore, this article will systematically review the roles of different types of programmed cell death in myocardial fibrosis and explore potential treatment methods based on these mechanisms.

## 1 Introduction

Cardiac fibrosis (CF) refers to the excessive deposition of extracellular matrix (ECM) proteins primarily composed of collagen fibers in the interstitium of the heart. As a common pathophysiological basis of various heart diseases—such as myocardial infarction, hypertrophic cardiomyopathy, diabetic cardiomyopathy, and dilated cardiomyopathy—it is closely associated with the occurrence of heart failure and malignant arrhythmia (1). Under normal conditions, cardiac fibroblasts, cardiomyocytes, endothelial cells, and vascular smooth muscle cells jointly maintain the homeostasis of the cardiac interstitium. In fibrotic diseases, resident fibroblasts in the heart are activated into myofibroblasts (MyoFBs), which exhibit the characteristics of high proliferation, massive synthesis of ECM proteins, and high expression of contractile proteins such as α-smooth muscle actin (α-SMA). This activation represents a core link leading to poor cardiac adaptive remodeling and progressive cardiac function decline (2).

Based on different pathological features, CF can be divided into two types: interstitial (diffuse) fibrosis and vicarious fibrosis (scar fibrosis) (3). Interstitial fibrosis is more common in hypertension, aortic stenosis, and various cardiomyopathies. It is characterized by diffuse deposition of collagen in the myocardial interstitium without obvious myocardial cell necrosis. Vicarious fibrosis occurs after myocardial infarction or severe myocardial injury, where cardiac fibroblasts proliferate to form scar tissue to replace necrotic myocardium. Although this process maintains structural integrity in the acute phase, it significantly reduces cardiac compliance and induces heart failure in the long term (4, 5). Due to the progressive and irreversible tendency of CF, current clinical treatment methods are limited to delaying the progression of the disease course. Effective intervention strategies targeting the etiology remain lacking, underscoring the urgent need to explore new molecular mechanisms and therapeutic targets.

PCD is a common process in organisms and is crucial for development, maintenance of cellular homeostasis, immunity, and stress response. The concept was first proposed by American biologists Richard A. Lockshin and Carroll M. Williams in 1964 to describe the cell’s self-clearance mechanism at specific spatiotemporal points during development (6). With advancing research, the scope of PCD has expanded, encompassing various forms such as classical apoptosis, necroptosis, autophagy, pyroptosis, and ferroptosis. These cell death patterns play important physiological roles in maintaining myocardial tissue homeostasis, eliminating damaged cells, and regulating the inflammatory response. Under pathological conditions, abnormal activation and imbalance of PCD can act as a condition driving CF. These death patterns can exert dual effects in CF: On the one hand, excessive or abnormal cardiomyocyte death directly releases damage-associated molecular patterns (DAMPs), activates the innate immune response and pro-fibrotic signaling pathways, indirectly induces the activation of cardiac fibroblasts and promotes ECM deposition, ultimately leading to CF. On the other hand, appropriate regulation of specific PCD pathways helps eliminate damaged or functionally abnormal cells, suppress the inflammatory response, create conditions for cell growth and tissue repair, and maintain the internal homeostasis of cardiac tissue, thereby delaying the progression of fibrosis. There are significant differences in the mechanisms of different PCD types in CF. Apoptosis is mainly triggered through the mitochondrial pathway or the death receptor pathway, and key regulatory factors such as Bcl-2 family proteins and caspase enzymes are abnormally expressed in fibrosis models (7). Autophagy has a bidirectional effect: Moderate autophagy can clear misfolded proteins and damaged organelles, while excessive autophagy promotes the activation of cardiac fibroblasts by degrading anti-fibrotic proteins (such as Beclin-1) (8). The core execution protein of pyroptosis—Gasdermin D—is significantly upregulated in myocarditis-related fibrosis, and the IL-18 released through its pore formation can induce the differentiation of cardiac fibroblasts into myofibroblasts (9). In addition, TGF-β, as the core regulator of CF, can promote the proliferation and differentiation of cardiac fibroblasts, as well as collagen maturation through Smad-dependent and non-Smad-dependent signaling cascades, playing a key role between PCD and fibrosis (10, 11).

Despite significant progress in researching the mechanisms of PCD in myocardial fibrosis, existing reviews primarily focus on a single type of cell death, lacking a systematic analysis of cross-regulation among multiple types and their therapeutic translation. Therefore, the present review aims to integrate the research advances of the past decade and provide a systematic account of the molecular mechanisms, pathophysiological roles, and therapeutic targets of different types of programmed cell death (PCD) in myocardial fibrosis. By comparing the signaling pathways and key regulatory factors of each death mode, along with their associations with fibrosis, and combining data from animal models and preclinical studies, we examine the potential and challenges of novel therapeutic strategies targeting PCD to reverse myocardial remodeling. Ultimately, it provides a theoretical basis for the development of precision anti-fibrotic therapies based on cell death regulation.

## 2 Pathogenesis of myocardial fibrosis

Myocardial fibrosis constitutes a critical pathological basis for the development of cardiovascular diseases (CVDs). Its essence lies in the pathological activation, proliferation, and differentiation of fibroblasts into myofibroblasts. These myofibroblasts mediate excessive synthesis and abnormal deposition of the ECM, primarily composed of collagen. Ultimately, this leads to a substantial accumulation of collagen fibers in the myocardial interstitium and a significant increase in ECM components (12). In particular, this is characterized by increased myocardial stiffness, impaired coupling of systolic–diastolic function, and abnormal electrical conduction. Fibrosis is a common end-stage feature of all types of cardiovascular diseases, including heart failure (HF), arrhythmia, and myocardial infarction (13). This condition is characterized by intractability and complexity, and its pathological mechanism has not been fully elucidated. Currently, no specific clinical treatment methods are available to reverse fibrosis and the treatment cost is high, thus posing a major clinical challenge. The pathogenesis of this disease involves a complex network of multi-cell interactions and multiple signaling pathways. This article will systematically analyze the core pathogenesis of myocardial fibrosis from the following aspects.

### 2.1 Epigenetic regulation

The essence of epigenetics resides in modifications at the chromatin level. Gene expression is regulated through chemical alterations of DNA and the histones that encase it, without any changes to the DNA sequence. Epigenetic regulation has been established as a vital factor in the pathogenesis of myocardial fibrosis. It influences the activation of cardiac fibroblasts, the expression of fibrotic genes, and the deposition of extracellular matrix during myocardial fibrosis by modulating DNA and modifying histones, among other mechanisms (14). There is a close relationship between the cellular metabolic state and epigenetic modifications. Recent studies have found that ATP citrate lyase (ACLY)—a key enzyme linking glucose metabolism and histone acetylation—is significantly upregulated in fibrotic heart tissue. ACLY catalyzes the cleavage of citrate to produce acetyl-CoA, providing an acetyl group donor for histone acetylation. The nuclear translocation of ACLY can increase pro-fibrotic gene promoter levels, thereby activating the transcriptional program of myofibroblasts. Inhibition of ACLY can reverse the phenotypic transformation of myofibroblasts by blocking the acetyl group supply (15).

Epigenetic regulation in CF is highly cell-specific. Taking histone acetylation as an example, the study by Zhang et al. (16) found that inhibiting KAT8-mediated H4K16ac can downregulate GATA4, which in turn reduces the expression of cardiomyocyte hypertrophy markers (cTnI, β-MHC) and indirectly improves the fibrotic microenvironment by alleviating cardiomyocyte stress. However, it is worth noting that GATA4 has different effects in fibroblasts. Mathison et al. (17) demonstrated that GATA4 in fibroblasts directly inhibits the pro-fibrotic transcription factor Snail; its deficiency leads to fibroblast senescence and senescence-associated secretory phenotype (SASP)-mediated fibrosis. Therefore, therapeutic strategies targeting the KAT8/GATA4 axis must take this complex bidirectional regulatory effect into account. In contrast, the deacetylase Sirtuins family (SIRT1–SIRT7) establishes an epigenetic defense mechanism against fibrosis. SIRT1 directly inhibits the expression of long non-coding RNA Neat1 at the transcriptional level by removing the acetylation modification of histone H3K27 (H3K27ac). As Neat1 is a key molecule driving the polarization of macrophages toward the pro-inflammatory M1 phenotype and assembly of inflammasomes, SIRT1-mediated inhibitory effect blocks pro-inflammatory and pro-fibrotic signals (such as IL-1β and TNF-α) transmitted from immune cells to fibroblasts, thereby inhibiting inflammation-driven fibroblast activation. Similarly, SIRT6 blocks the maturation and processing of pro-fibrotic microRNAs (miR-216/217) by reducing H3K56ac levels. By relieving degradation of downstream anti-fibrotic target genes by miRNAs, the proliferation and collagen synthesis of fibroblasts are inhibited (18). Collectively, these findings highlight a multidimensional regulatory network involving the metabolism–immunity–epigenetics axis in the occurrence and development of CF. In addition, the abnormal expression of non-coding miRNAs is also crucial in myocardial fibrosis. miRNAs are a class of single-stranded, non-coding RNA molecules approximately 22 nucleotides in length, which are involved in the post-transcriptional regulation of genes related to various biological processes. In cardiac fibrosis, they can be classified into pro-fibrotic miRNAs (FibromiRs) and anti-fibrotic miRNAs (19). MiRNAs are widely involved in the regulation of cardiac remodeling after different CVDs (20, 21) functioning by binding to the mRNA of target genes and inhibiting protein translation to regulate the expression of specific proteins (22). Clinical data indicate that in the myocardial tissues of heart failure patients, there is often an upregulation of pro-fibrotic miR-21 and miR-208a and a downregulation of anti-fibrotic miR-29 (23). miR-21 is a core driver of fibrosis. On the one hand, it targets and inhibits Sprouty-1, relieving the inhibition of the extracellular signal-regulated kinase (ERK)–mitogen-activated protein (MAP) pathway in cardiac fibroblasts. On the other hand, by inhibiting the expression of dual-specificity phosphatase 8, it continuously activates the c-Jun N-terminal kinase (JNK) and p38 MAPK signaling axes. This dual kinase activation mechanism further promotes the phosphorylation and nuclear translocation of pro-fibrotic transcription factors such as activator protein 1 (AP-1) and ETS-like protein 1 (Elk-1), initiating the transcription program of type I and type III collagen genes and significantly increasing the proliferation of fibroblasts and the excessive deposition of ECM (24, 25). In terms of anti-fibrosis, miR-378 released by cardiomyocytes exerts an anti-fibrotic effect by targeting mitogen-activated protein kinase kinase 6 (MKK6) in cardiac fibroblasts and reducing the phosphorylation of p38 MAPK (26). The extract of Pericarpium Trichosanthis can specifically upregulate the expression of anti-fibrotic miR-29b, thereby blocking the transmission of the transforming growth factor-β1 (TGF-β1)/mothers against the decapentaplegic homolog 3 (Smad3) signaling pathway and significantly alleviating the degree of CF in heart failure rats (27). This further demonstrates that pro-fibrotic and anti-fibrotic related miRNAs play important roles in CF. Long non-coding RNAs (lncRNAs)—acting as signal molecules (Signal), decoy molecules (Decoy), guide molecules (Guide), or scaffold molecules (Scaffold)—play crucial regulatory roles in the signaling cascade of cardiac fibrosis through extensive interactions with DNA, RNA, and proteins (28). Although the research on lncRNAs in CF is still in the exploratory stage, existing studies have revealed their unique mechanisms. Ge et al. (29) reported that the expression of lncRNA Neat1 was upregulated in the myocardial tissues of mice undergoing aortic constriction surgery and in cardiac fibroblasts treated with TGF-β1. It promotes the occurrence and development of CF and exacerbates cardiac function damage by binding to EZH2 and inhibiting the expression of Smad7. lncRNAs can also function as competitive endogenous RNAs (ceRNAs). Wang et al. (30) found that lncRNA554 was significantly upregulated after myocardial infarction. By sponging miR-543, it relieves the inhibition of TSP-1, thereby activating the TGF-β1/Smad3 signaling pathway. Knockdown of lncRNA can significantly improve the fibrotic phenotype. At present, the newly discovered THBS1-AS1 directly drives fibroblast activation by specifically enhancing the mRNA stability of TGF-β type I receptor (TGFBR1) (31). Circular RNAs (circRNAs) are also involved in regulation through complex mechanisms. Research has demonstrated that under diabetic and Ang II-induced conditions, there is a significant increase in the expression of circRNA_000203. This molecule has been shown to bind to microRNA-26b-5p, thereby blocking the post-transcriptional repression of genes encoding extracellular matrix components, such as collagen 1A2 and connective tissue growth factor (CTGF), which are otherwise suppressed by this microRNA. Although CTGF expression is markedly elevated in this pathological process, recent clinical research advances predominantly view it as a biomarker, reflecting changes in the fibrotic microenvironment rather than a single core therapeutic target. This imbalance in the non-coding RNA regulatory network ultimately accelerates the pathological progression of cystic fibrosis (32). Finally, RNA chemical modification (epitranscriptomics), as an emerging mechanism, has been receiving much attention. In addition to the regulation of non-coding RNAs, the chemical modification of mRNA itself directly determines the development of fibrosis. The latest research reveals that the acetyltransferase NAT10 can mediate the N4-acetylcytosine (ac4C) modification of TGFBR1 mRNA. This modification significantly enhances the stability and translation efficiency of TGFBR1 mRNA, leading to an increase in the density of cell surface receptors and continuous amplification of TGF-β signaling (33). This discovery not only reveals a new pathogenic mechanism but also suggests that targeting RNA modification enzymes may represent a new strategy for treating CF. In summary, epigenetic regulation plays multiple roles in the pathogenesis of CF through histone modification and non-coding RNA networks. By regulating the transcription and expression of key genes involved in fibrosis, it affects fibroblast activation, macrophage polarization, and intercellular crosstalk mechanisms, thereby influencing the process of fibrosis.

### 2.2 Renin–angiotensin–aldosterone system

The renin­–angiotensin–aldosterone system (RAAS) plays a crucial role in the occurrence and development of cardiac fibrosis. After cardiac injury, pro-fibrotic mediators, including RAAS components, activate the differentiation of fibroblasts, leading to the proliferation and migration of myofibroblasts and the deposition of ECM proteins, thereby resulting in the formation of fibrosis (34). Angiotensin II (Ang II) is a key effector molecule of RAAS. After cardiac injury, macrophages migrate to the injured area and induce the activation of fibroblasts into myofibroblasts, produce renin and angiotensin-converting enzyme (ACE), and promote CF through multiple pathways. Studies have shown that Ang II directly promotes the proliferation of cardiac fibroblasts, inhibits fibroblast migration, promotes integrin expression, and facilitates the transformation of myofibroblasts by activating the angiotensin II type 1 receptor (AT1R), significantly increasing the excessive deposition of the ECM. Among them, the abnormal accumulation of type I and type III collagen is a typical feature of CF. In addition, Ang II can also activate a series of intracellular signaling pathways and enhance the tissue inflammatory response, leading to the upregulation of transforming growth factor-β (TGF-β), interleukin-1β (IL-1β), and tumor necrosis factor-α (TNF-α) expression, which further promotes the activation of fibroblasts and the expression of fibrosis-related genes (35, 36). After binding to the widely expressed AT1R, Ang II can activate G protein-coupled signaling and trigger the RAS–RAF­–MEK–ERK signaling cascade, leading to the phosphorylation of mitogen-activated protein kinase (MEK) and ERK. Ultimately, this contributes to the development of CF. This remodeling process is frequently accompanied by a significant upregulation of fibrosis markers, such as CTGF (37, 38). In the study by Lei et al. (39), it was found that after stimulation with Ang II, salvianolic acid (DSS) can specifically inhibit the phosphorylation of ERK2 at Thr188, thereby interfering with the homodimerization, nuclear translocation of ERK2, and the activation of downstream pro-fibrotic signal-induced CF formation. In addition, Ang II can cause the production of reactive oxygen species (ROS). ROS triggers the uncoupling of endothelial nitric oxide synthase, reduces the bioavailability of nitric oxide (NO), and upregulates the expression of intercellular adhesion molecule-1 and vascular cell adhesion molecule-1, promoting vascular inflammatory responses. This induces the activation of the pro-fibrotic TGF-β1-Smad2/3 signaling pathway, the synthesis of type I and type III collagen, and the trans-differentiation of cardiac fibroblasts into myofibroblasts. ROS can further activate pathways such as ERK, JNK, and p38 MAPK, which jointly regulate the proliferation and survival of fibroblasts and the expression of collagen, participating in the formation of CF (40, 41). Current preclinical experiments demonstrate that the use of RAAS inhibitors [such as ACE inhibitors or angiotensin receptor blockers (ARBs)] can significantly reduce TGF-β expression, decrease collagen deposition, and improve cardiac function (42). In animal models, treatment with ARBs (such as losartan) and ACE inhibitors (such as enalapril) can block the Ang II-mediated TGF-β signaling pathway, significantly reducing collagen deposition and myocardial ECM remodeling, even when blood pressure does not change significantly in some cases, further supporting the hemodynamic independence of their anti-fibrotic effects (43). In conclusion, the renin–angiotensin–aldosterone system (RAAS) plays a central regulatory role in cardiac fibrosis. It can regulate the blood pressure system and integrate a complex molecular network involving mechanical stress conduction, immune microenvironment remodeling, and oxidative stress regulation. Through direct intranuclear transcriptional reprogramming (such as activation of the ERK-mediated core pro-fibrotic gene cluster) and indirect paracrine/oxidative modification (the ROS/TGF–*β*-axis), it constructs a vicious cycle of fibrosis. The non-hemodynamic-dependent anti-fibrotic effects confirmed by clinical and experimental evidence not only consolidate the central position of RAAS blockers in reversing cardiac remodeling but also highlight the need for in-depth exploration of downstream signaling nodes (such as targeting specific phosphorylation sites of ERK2 or specific ROS subtypes), which may open new avenues for developing more precise and safer anti-fibrotic targeted drugs.

### 2.3 TGF-β signaling pathway

The TGF-β signaling pathway is a core factor inducing myocardial fibrosis (44). Multiple forms of programmed cell death jointly promote the activation of pro-fibrotic signals by altering the local inflammatory and repair microenvironment in the myocardium. Among these, the TGF-β signaling pathway is considered the core downstream signal in PCD-mediated CF. Basic research has shown that DAMPs, inflammatory cytokines, and oxidative stress signals released during cardiomyocyte apoptosis, pyroptosis, or ferroptosis can directly or indirectly activate the expression of TGF-β1, thereby driving the phenotypic transformation of fibroblasts into myofibroblasts (45). TGF-β1 remodels transcriptional profiles of fibroblasts through the classical Smad-dependent pathway. After TGF-β binds to the type II receptor complex on the cell membrane, it recruits and phosphorylates the type I receptor, inducing the phosphorylation of receptor-regulated transcription factors Smad2 and Smad3. Once phosphorylated, they act as transcription factors to upregulate the expression of type I and III collagens, fibronectin 1, α2-actin, and periostin in the nucleus, increasing the contractility and secretory function of cells, marking their transformation into myofibroblasts (46). TGF-β also increases the deposition of collagens I, III, and VI and enhances the expression of matrix proteins in myofibroblasts by regulating the expression of plasminogen activator inhibitors, tissue inhibitors of metalloproteinases, and pro-fibrotic cytokines (47). Basic research has shown that in metabolic heart diseases such as diabetic cardiomyopathy, TGF-β accelerates the CF process by activating the Smad-dependent transcriptional program, promoting the activation of cardiac fibroblasts and ECM deposition (48). In a myocardial infarction model, inhibiting the TGF-β1/Smad signal can significantly reduce myocardial interstitial collagen deposition, suggesting that this pathway plays a crucial role in post-injury fibrotic remodeling (49). Similarly, in a hypertensive heart failure model, interventions targeting the TGF-β1/Smad pathway can improve the degree of CF and restore cardiac function (50), further demonstrating the role of this signaling axis in CF of different etiologies. Rao et al. (51) demonstrated that Xin-Ke-Shu may prevent CF by inhibiting the TGF-β1 pathway and reducing MMP expression. Therefore, the TGF-β/Smad pathway is not only a classic regulator*y* axis of CF but also a key molecular hub for the transformation of multiple programmed cell death signals into structural cardiac remodeling.

### 2.4 Cardiac fibroblasts and myofibroblasts

Under physiological conditions, cardiac fibroblasts remain in a relatively quiescent state. They maintain the dynamic homeostasis of the ECM by regulating the synthesis and degradation of type I and type III collagens, fibronectin, and laminin. Among them, type I and type III collagens account for approximately 85% and 10% of total collagen, respectively, jointly determining the structural support and mechanical properties of the myocardium (52). Cardiac fibroblasts also participate in the regulatory homeostasis of cardiomyocytes and endothelial cells by secreting paracrine factors, such as TGF-β, platelet-derived growth factor (PDGF), and vascular endothelial growth factor (53). Under pathological stimuli—such as oxidative stress, inflammatory response, excessive mechanical load, or persistent activation of neurohumoral factors—quiescent cardiac fibroblasts are rapidly activated, characterized by enhanced proliferative capacity and trans-differentiation into α-SMA-positive myofibroblasts. Myofibroblasts acquire significantly enhanced contractile phenotypes and secretory functions. Their continuous activation can lead to excessive synthesis, abnormal cross-linking, and impaired degradation of ECM proteins, thereby increasing tissue stiffness and forming a vicious cycle that promotes myocardial structural remodeling and functional deterioration (35, 54). Numerous studies highlight that the formation and maintenance of myofibroblasts are key determinants in the occurrence and progression of cardiac fibrosis. Moreover, myofibroblasts interact with cardiomyocytes, immune cells, and endothelial cells to form an intercellular regulatory network characterized by ECM remodeling and amplification of pro-fibrotic signals, thereby amplifying and sustaining the fibrotic response (55–57). In the early stage of the fibrotic reaction, some myofibroblasts may lack α-SMA expression yet still participate in ECM remodeling as myofibroblast-like cells. Regardless of the causes and triggering factors of CF, the continuous trans-differentiation of fibroblasts into myofibroblasts is widely regarded as a common pathological feature and core step in cardiac fibrotic reactions (58). Currently, several studies have also shown that inhibiting the transformation of cardiac fibroblasts into myofibroblasts plays an important role in CF. The traditional Chinese medicine compound Shexiang Baoxin Pill can significantly block the phenotypic transition of fibroblasts into myofibroblasts by inhibiting the phosphorylation of signal transducer and activator of transcription 3 (STAT3) in fibroblasts, thereby reducing the expression of α-SMA and collagen and improving myocardial interstitial fibrosis. This study suggests that the STAT3 signaling plays a crucial role in maintaining the activated state of myofibroblasts; its abnormal activation may constitute an important molecular basis for the continuous progression of CF (59). Moreover, the basic helix–loop–helix transcription factor TCF21 has been identified as an important endogenous inhibitor of fibroblast phenotypic homeostasis. Research indicates that TCF21 is highly expressed in quiescent cardiac fibroblasts but significantly downregulated in myofibroblasts. The loss of TCF21 can promote the trans-differentiation of fibroblasts into α-SMA-positive myofibroblasts and exacerbate cardiac fibrosis, whereas maintaining or restoring its expression can inhibit myofibroblast formation and alleviate the degree of CF (60). These studies further emphasize—at the transcriptional regulation level—that the trans-differentiation of fibroblasts into myofibroblasts is not a one-way, irreversible process but a dynamic, plastic event finely regulated by multilevel signaling networks. Although multiple signaling pathways and molecular regulatory networks involved in this process have been identified, clinically effective treatment strategies that can directly and specifically block or reverse the abnormal activation of myofibroblasts are still lacking, making this process an important target in anti-CF research.

## 3 A discourse on the mechanisms of programmed cell death in myocardial fibrosis

Cell death is a fundamental life process that maintains tissue homeostasis and responds to internal and external environmental stresses. When cells experience irreversible damage—such as DNA damage, oxidative stress, or metabolic disorders—and the cell cycle arrest and damage repair mechanisms fail to restore homeostasis, relevant pathways such as PCD will be activated. As a mechanism for precisely regulating cell fate, PCD plays a key role in growth and development, maintaining intracellular environmental homeostasis, immune regulation, and the ability to respond to external environmental stimuli (61). According to different mechanisms such as morphological characteristics, immunology, and genetic features, PCD can be divided into multiple forms, including apoptosis, autophagy, necroptosis, pyroptosis, and ferroptosis (62, 63). Increasing evidence indicates that these different types of PCD are intertwined under pathological cardiac conditions, jointly shaping the inflammatory microenvironment and remodeling tissue after myocardial injury. PCD plays an important role in maintaining the homeostasis of cardiomyocytes and has a dual impact on cardiac fibrosis. Moderate cell death helps remove damaged cells and promote tissue reconstruction, while excessive or continuously activated PCD significantly amplifies myocardial injury and accelerates the fibrosis process by releasing DAMPs and inducing chronic aseptic inflammatory responses (64). Cardiomyocytes are terminally differentiated cells with extremely limited self-renewal ability, and their death often becomes an important factor in cardiac structural remodeling. In this context, various pro-inflammatory and pro-fibrotic signals released by dead or damaged cardiomyocytes can significantly activate cardiac fibroblasts, driving their proliferation and trans-differentiation into myofibroblasts, ultimately leading to the formation of cardiac fibroblasts to repair the injury (65). Therefore, systematically analyzing the roles of different types of PCD in the pathological mechanism of myocardial injury–fibroblast activation–fibrosis formation is of great importance. Such insights will deepen our understanding of the mechanism of CF and aid the discovery of potential drug intervention targets.

### 3.1 Apoptosis

Apoptosis is an energy-dependent form of programmed cell death mediated by the cascade reaction of the caspase family. Its morphological characteristics include cell shrinkage, chromatin condensation, and the formation of apoptotic bodies, typically not triggering a severe inflammatory response. As a typical form of programmed cell death, apoptosis is a core process for maintaining organismal homeostasis, regulating the entire processes of normal cell renewal, the immune system, tissue homeostasis, embryonic and brain development, and disease defense (66). At the molecular level, apoptosis is mainly executed through two pathways. The intrinsic pathway mainly involves mitochondrial dysfunction, and its activation conditions include various signals such as DNA damage and oxidative stress. Among them, the Bcl-2 family proteins are the core regulators of this pathway. Once the pro-apoptotic proteins Bax and Bak are activated, they form pores on the outer mitochondrial membrane, leading to the release of cytochrome *c*, which then binds to Apaf-1 to form the apoptosome. The apoptosome recruits and activates the initiator enzyme caspase-9, thus initiating the proteolytic process associated with cell death (67, 68). The extrinsic pathway is initiated by extracellular ligands such as FasL and TNF-α binding to cell surface death receptors like Fas and TNFR. After receptor aggregation, the adapter protein FADD is recruited, forming the death-inducing signaling complex and activating caspase-8, which results in the proteolytic cleavage of multiple substrates, thereby initiating the execution phase of apoptosis (69, 70). The activation of caspase-3 leads to the degradation of actin, nuclear lamin, and endonuclease inhibitors, thereby accelerating cell disintegration.

**Figure 1:**
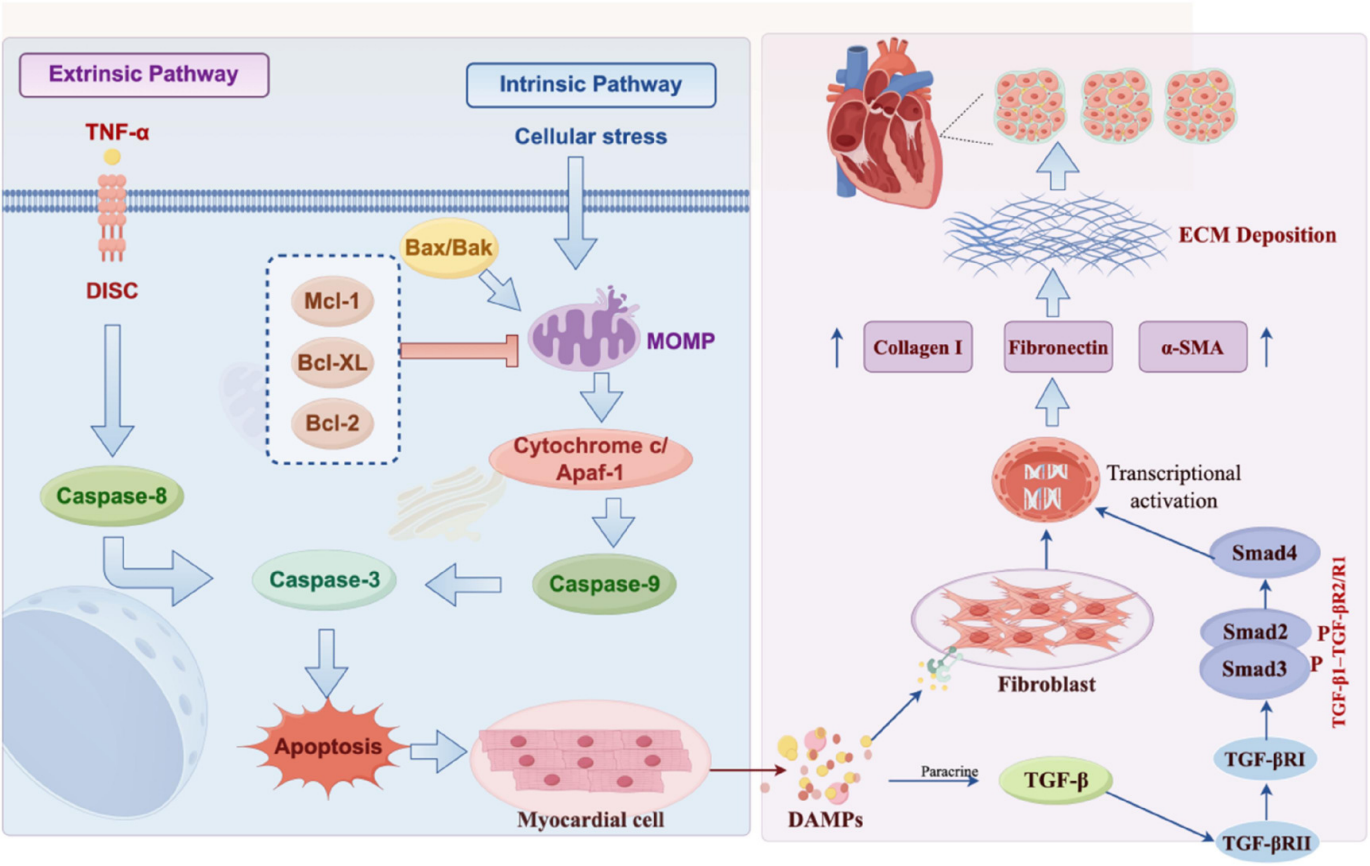
Apoptosis pathways and their role in cardiac fibrosis. (Left) The extrinsic pathway is activated by TNF-α and caspase-8. The intrinsic pathway is triggered by cell stress, mitochondrial damage (MOMP), cytochrome c release, and caspase-9 activation. Both pathways activate caspase-3, leading to cardiomyocyte apoptosis. (Right) Apoptotic cells release DAMPs, which activate fibroblasts to produce TGF-β. GF-β binds its receptors (TGF-βRI/II), activating Smad2/3/4 and promoting transcription of fibrotic genes. This leads to ECM deposition (collagen I, fibronectin, α-SMA) and myocardial fibrosis).

Recent studies have revealed that apoptosis drives the fibrotic process by mediating cardiomyocyte loss, fibroblast activation, and ECM remodeling (as shown in Figure 1). The underlying mechanisms involve multiple pathophysiological processes such as oxidative stress, endoplasmic reticulum (ER) stress, and inflammatory responses, regulated by multiple signaling pathways. Myocardial ischemia/reperfusion (I/R) injury is a classic model of cardiomyocyte apoptosis. During I/R, cardiomyocytes experience hypoxia/reoxygenation, which significantly upregulates the expression of the pro-apoptotic protein Bax and downregulates the expression of the anti-apoptotic protein Bcl-2, thereby activating the mitochondrial apoptotic pathway. Research has shown that the long non-coding RNA 1700020I14Rik is downregulated during I/R. As a ceRNA, it sponges miR-297a to protect the expression of its downstream target gene, calcitonin gene-related peptide (CGRP). CGRP not only inhibits cardiomyocyte apoptosis but also acts as a potent anti-fibrotic neuropeptide, directly inhibiting the proliferation and collagen synthesis of cardiac fibroblasts through paracrine signaling. Therefore, downregulation of this lncRNA leads to the loss of protective CGRP, relieving the inhibition of fibroblast activation and exacerbating post-injury fibrotic remodeling (71). In the doxorubicin (DOX)-induced injury model, miR-133b directly targets and inhibits the expression of polypyrimidine tract-binding protein 1 and transgelin 2 at the post-transcriptional level. These two target proteins normally participate in the cell death process as effectors of apoptosis and cytoskeleton disruption. Therefore, the overexpression of miR-133b significantly inhibits collagen accumulation and alleviates cardiac fibrosis *in vivo* by maintaining cytoskeletal integrity and blocking the mitochondrial apoptotic cascade (72). ER stress is another important inducer of cardiomyocyte apoptosis. Calreticulin is upregulated in the cardiac tissues of human ischemic heart failure, and its overexpression promotes ER stress-induced cardiomyocyte apoptosis, often manifesting as increased expression of apoptotic markers such as caspase-12 and CHOP (73). In addition, oxidative stress is a major driver of CF activation and apoptosis. Excessive production of ROS directly damages cardiomyocyte mitochondria, leading to a decrease in mitochondrial membrane potential and dysfunction, which in turn activates the mitochondrial-dependent apoptotic signaling pathway. In diabetic cardiomyopathy, metabolic disorders caused by hyperglycemia lead to excessive accumulation of mitochondrial ROS, inducing sustained oxidative stress state. This not only directly initiates the endogenous apoptosis program of cardiomyocytes by disrupting the mitochondrial membrane potential but also promotes the proliferation of cardiac fibroblasts and collagen deposition through the activation of specific signaling pathways, thereby exacerbating cardiac fibrosis (74). The natural polyphenolic flavonoid scutellarin has been proven to alleviate doxorubicin (DOX)-induced chronic cardiotoxicity. Scutellarin (SCU) can significantly reduce the level of oxidative stress and cardiac troponin T (cTnT) in myocardial tissue. Meanwhile, it reduces ECM accumulation and improves CF by inhibiting the TGF-*β*1/Smad2 signaling pathway, while inhibiting apoptosis by upregulating Bcl-2 and downregulating Bax and cleaved caspase-3 (75). Similarly, in an experimental study on the cardiotoxicity of the organophosphorus flame-retardant triphenyl phosphate (TPHP), TPHP promotes mitophagy through mitochondrial fusion dysfunction caused by oxidative stress; upregulates the expression levels of Bax, cleaved caspase-9, and cleaved caspase-3; and downregulates the expression level of Bcl-2 in myocardial tissue, leading to cardiomyocyte apoptosis and fibrosis (76). In a porcine model of chronic coronary ischemia, the DPP-4 inhibitor linagliptin demonstrates a dual protective mechanism. By downregulating factors such as apoptosis-inducing AIF, pro-apoptotic protein Bad, and caspase-9, it significantly reduces the level of TGF-β in tissues and improves the survival of cardiomyocytes (77). In the treatment of acute heart failure, the intervention of relaxin not only inhibits TGF-β1/α-SMA-mediated type I collagen deposition but also reverses the decrease and lateral distribution of connexin 43 (Cx43) in the infarct border zone. Since the disorder of Cx43 is usually triggered by apoptotic signals and leads to dual remodeling of electrical and structural properties, this effect of relaxin suggests that by inhibiting apoptosis, it can simultaneously improve cardiac fibrosis and electrophysiological stability (78). In conclusion, cardiomyocyte apoptosis is not only the endpoint of myocardial injury but also the starting point of fibrosis initiation. Blocking the conversion of apoptotic signals to pro-fibrotic signals is the key to improving myocardial remodeling. However, while apoptosis is generally considered to be an immunologically “silent” process, it has been demonstrated that extensive cell death and the subsequent release of cellular contents can trigger intense inflammatory responses. This transition from silent clearance to inflammatory amplification signifies the emergence of another critical PCD form: pyroptosis.

The prevention of the conversion of apoptotic signals to pro-fibrotic signals is a critical component of enhancing myocardial remodeling.

### 3.2 Pyroptosis

**Figure 2:**
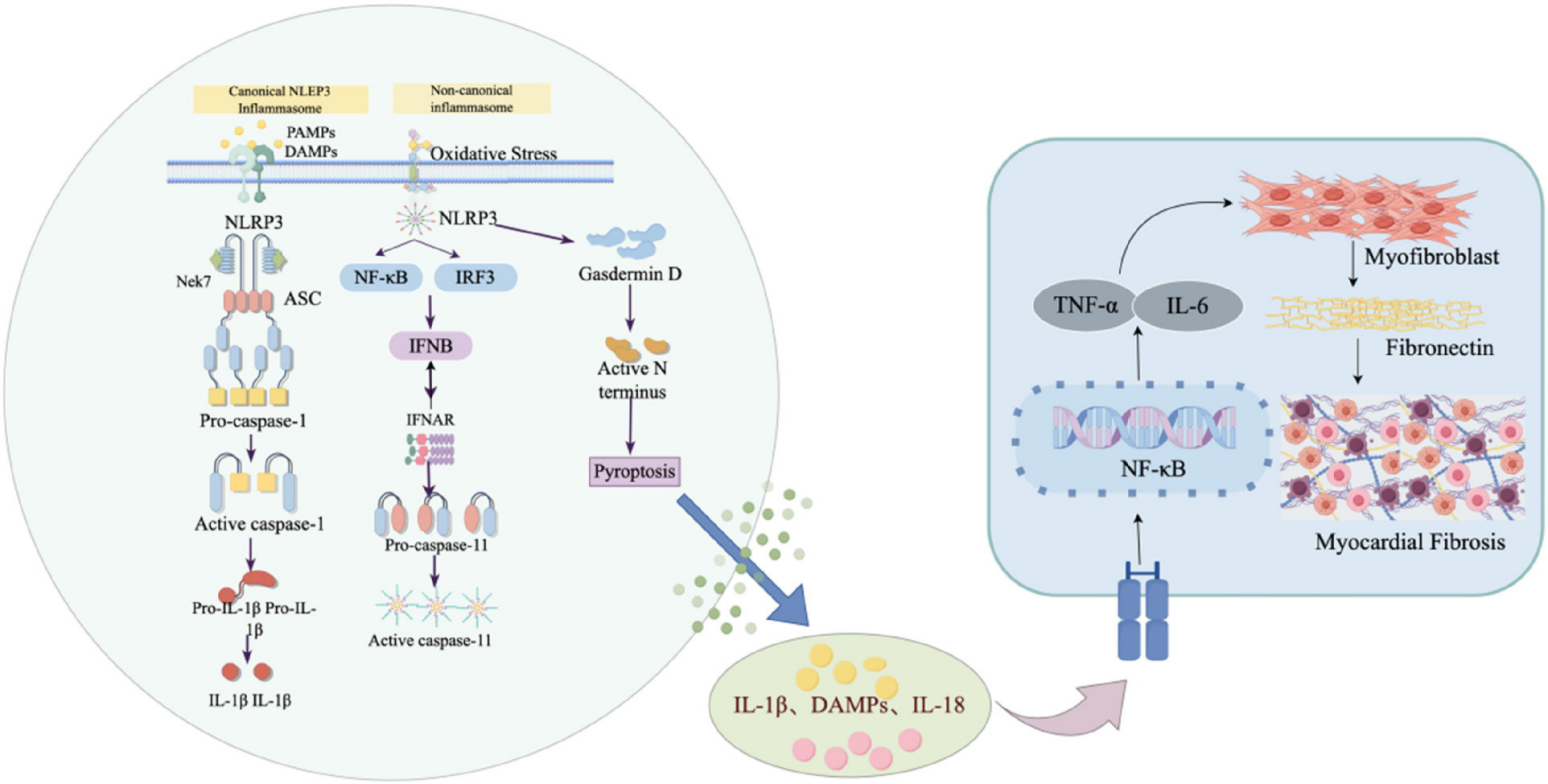
The mechanism of pyroptosis in myocardial fibrosis. (Left) Both canonical (NLRP3/caspase-1) and non-canonical (caspase-11) inflammasome pathways are shown. Activation by PAMPs/DAMPs or oxidative stress leads to inflammasome assembly and cleavage of gasdermin D, forming membrane pores that trigger pyroptosis. This process also promotes release of pro-inflammatory cytokines IL-1β, IL-18, and DAMPs. (Right) The released mediators activate NF-κB signaling, upregulating TNF-α and IL-6, which promote fibroblast-to-myofibroblast transition and expression of fibronectin, resulting in myocardial fibrosis.

Among the various forms of programmed cell death, pyroptosis is regarded as a crucial link between myocardial injury and fibrotic remodeling due to its potent inflammation-amplifying effect. Pyroptosis is a form of programmed cell death characterized by inflammation amplification, primarily mediated by the activation of the NLRP3 inflammasome. Its typical features include the disruption of cell membrane integrity, the release of intracellular contents, and the secretion of large amounts of pro-inflammatory cytokines such as IL-1β and IL-18 (79). The canonical pyroptotic pathway relies on the activation of caspase-1, which cleaves Gasdermin D (GSDMD) to generate its N-terminal active fragment (NT-GSDMD), forming non-selective pores in the cell membrane, ultimately leading to cell swelling, rupture, and the release of inflammatory factors (80). In addition, the non-canonical pyroptotic pathway can be mediated by caspase-4/5 (in humans) or caspase-11 (in mice). The activation of caspase-3 or caspase-8 can also participate in regulation under specific pathological conditions, thus expanding the biological role of pyroptosis in different disease contexts (81, 82). These released inflammatory mediators not only directly exacerbate local myocardial inflammatory response but also initiate and activate cardiac fibroblasts, promoting their transformation into myofibroblasts and the abnormal secretion of collagen, thereby driving the abnormal proliferation of fibrous tissue (83). Numerous studies have demonstrated that pyroptosis plays a crucial role in the cardiac fibrosis process associated with various cardiovascular diseases, including hypertension, myocarditis, diabetic cardiomyopathy, myocardial ischemia–reperfusion injury, and metabolic disorders. Under the aforementioned pathological conditions, after myocardial cells are stimulated by oxidative stress, mechanical stretch, or DAMPs, the NLRP3 inflammasome is activated, and it recruits and cleaves pro-caspase-1 through ASC. On the one hand, activated caspase-1 cleaves GSDMD, inducing pyroptosis in myocardial cells and causing damage to the cardiac structure; on the other hand, it promotes the maturation and extracellular secretion of pro-IL-1β and pro-IL-18, driving the local inflammation amplification reaction, promoting fibroblast activation, and ECM deposition, ultimately accelerating the cardiac fibrosis process (84, 85) (as shown in Figure 2). Therefore, the core of the pyroptosis–inflammation–fibrosis axis lies in the persistent inflammation activation mediated by the NLRP3 inflammasome, which provides a pro-inflammatory microenvironment for CF activation and the initiation of the fibrosis program by amplifying the local immune response. Studies have shown that the classic drug metformin can activate and phosphorylate AMPK, reduce the release of pro-inflammatory cytokines (TNF-α, IL-6, IL-1β), inhibit the activation of the NLRP3 inflammasome, significantly reduce the myocardial infarct size and pyroptosis, and thus inhibit the occurrence of cardiac fibrosis (86). Similarly, Zhang et al. (87) demonstrated in a heart failure (HF) model of doxorubicin (DOX)-induced myocardial injury in mice that MCC950 inhibits the NLRP3 inflammasome-mediated pyroptotic pathway by inhibiting the activation of the NLRP3 inflammasome, improving the inflammation level and alleviating CF. In an isoproterenol-induced cardiac fibrosis model, NLRP3 deficiency significantly reduced myocardial collagen deposition and the expression of inflammatory factors. The mechanism is related to the fact that NLRP3 deficiency inhibits the formation of GSDMD membrane pores, limiting the release of high-mobility group box 1 (HMGB1). As HMGB1 induces fibroblast transformation through the TLR4 receptor, blocking the ROS/HMGB1 signaling axis significantly inhibits the progression of CF. Natural products such as salidroside and leonurine can block the pro-fibrotic cascade reaction triggered by myocardial cell pyroptosis by inhibiting the TLR4 pathway or regulating the TGF-β/Smad2 signaling pathway (88, 89). In addition, excessive mitochondrial reactive oxygen species (mtROS) generated by mitochondrial dysfunction are considered a core danger signal for the activation of the NLRP3 inflammasome. Liu et al. (90) demonstrated that hydrogen sulfide (H_₂_S) can inhibit the activation of the NLRP3 pathway by scavenging ROS, improve myocardial cell pyroptosis in diabetic cardiomyopathy, and thus alleviate CF. Further mechanistic studies have shown that the mitochondria-targeted H_₂_S donor AP39 can specifically activate the AMPK-ULK1-FUNDC1-mediated mitophagy process. Enhanced mitophagy flux reduces the ROS level, improves the mitochondrial membrane potential, inhibits the activation of the NLRP3 inflammasome, and significantly alleviates CF (91). These findings collectively reveal that pyroptosis can promote the occurrence of CF through mechanisms such as direct myocardial injury, the release of pro-inflammatory factors, and mitophagy, while the fibrotic microenvironment in turn exacerbates pyroptosis, forming a vicious cycle. Therefore, multi-targeted intervention strategies directed at the NLRP3/caspase-1/GSDMD axis and its upstream metabolic and mitochondrial homeostasis regulation may provide more translational potential therapeutic approaches for blocking the vicious pyroptosis–inflammation–fibrosis cycle. In order to counteract such inflammatory damage and maintain intracellular homeostasis, cardiomyocytes activate a conserved degradation mechanism that clears damaged organelles (such as mitochondria) and misfolded protein aggregates. This protective intracellular degradation process is known as autophagy.

### 3.3 Autophagy

**Figure 3:**
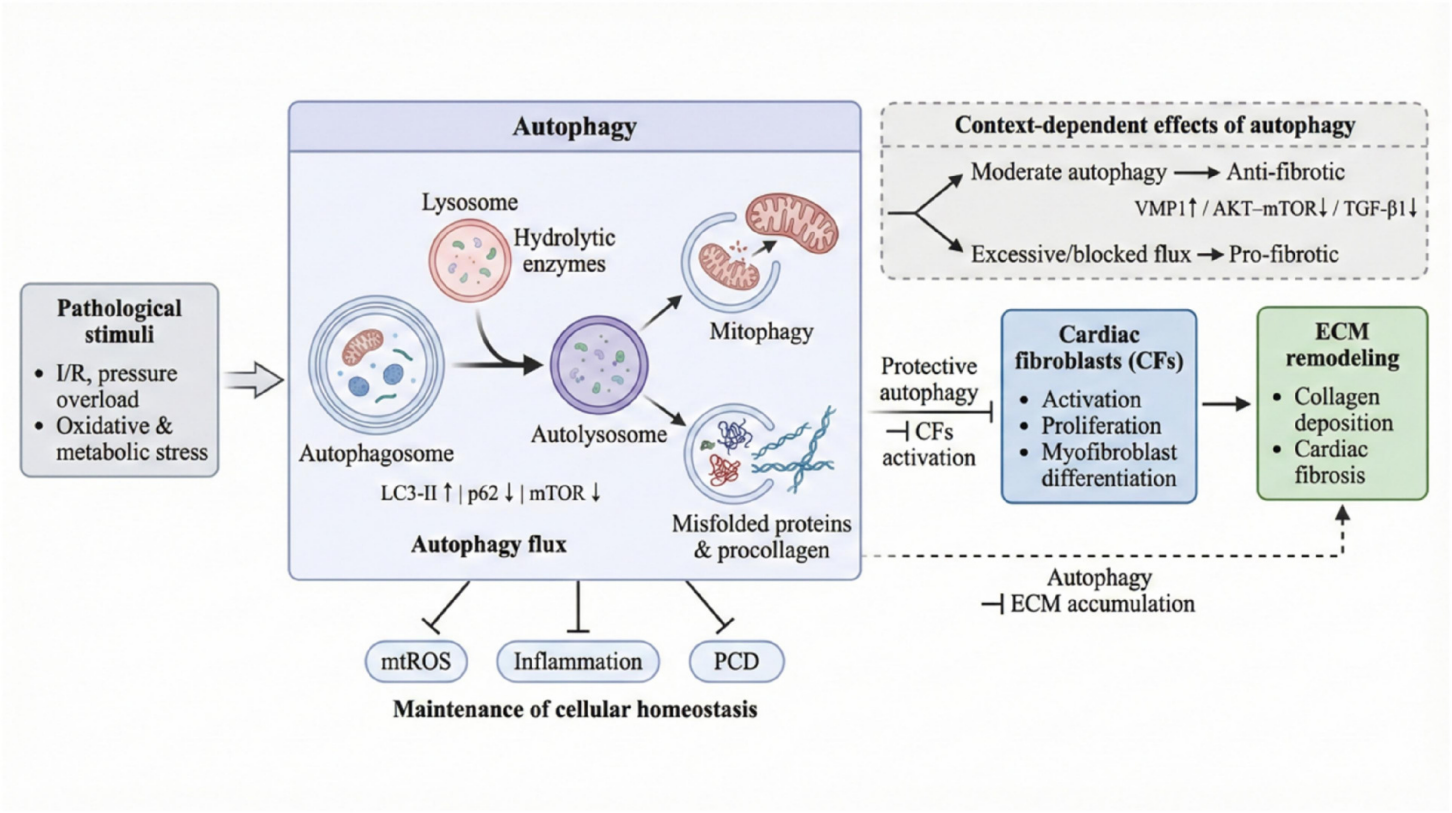
The mechanism of autophagy in myocardial fibrosis. Dual roles of autophagy in fibrosis. **(A)** Anti-fibrotic: functional autophagy inhibits signaling by degrading TGF-β type II receptors (TGF-β RII) and limits ROS via PINK1/Parkin-mediated mitophagy. **(B)** Pro-fibrotic: dysregulation promotes fibrosis through excessive degradation causing cell death, or flux blockade (by HSPA9/DNMT3A) leading to the accumulation of pro-fibrotic factors.

Autophagy is a highly conserved intracellular degradation and recycling mechanism that maintains intracellular homeostasis by forming autophagosomes and transporting damaged organelles, misfolded proteins, and other macromolecules to lysosomes for degradation. In cardiac tissues, autophagy is of great significance in maintaining myocardial homeostasis, buffering metabolic and oxidative stress, and limiting programmed cell death. Autophagy also plays a crucial regulatory role in the activation, proliferation, migration, and trans-differentiation of cardiac fibroblasts into myofibroblasts. Autophagy regulation has a dual effect. In the physiological or compensatory stage, moderate autophagy exhibits anti-fibrotic effects. Autophagy reduces mtROS levels and inhibits apoptosis and inflammatory responses by removing damaged mitochondria and harmful protein aggregates generated under ischemia, oxidative stress, or pressure overload conditions, thereby reducing myocardial cell death and replacement fibrosis (92, 93). In addition, autophagy may also be involved in the maintenance of ECM homeostasis. By degrading the intracellularly over-synthesized or misfolded procollagen, it restricts its abnormal secretion, thus inhibiting the fibrotic process (94) (as shown in Figure 3). In I/R injury, the endoplasmic reticulum protein VMP1 can effectively inhibit the activation of cardiac fibroblasts and the deposition of collagen I induced by TGF-β1 and PDGF-BB by enhancing autophagic flux. This is evidenced by an increase in the LC3-II/LC3-I ratio, p62 degradation, and inhibition of mTOR phosphorylation (95). Similarly, the natural compound plumbagin upregulates the autophagy level by inhibiting the AKT/mTOR pathway, thereby suppressing the fibrosis of TGF-β1-induced cardiac fibroblasts (96). These findings suggest that enhancing autophagy can serve as a protective mechanism to limit the pathological activation of cardiac fibroblasts and the fibrotic process. Conversely, under abnormal conditions—such as excessive autophagy activation or impaired autophagic flux—autophagy may also exacerbate fibrosis by promoting CF activation and ECM accumulation. In cardiac fibroblasts, autophagy can participate in regulating activation and differentiation through multiple pro-fibrotic signaling pathways—such as the TGF-β, PI3K/Akt/mTOR, and PINK1/Parkin—limit the excessive transformation of cardiac fibroblasts into myofibroblasts, and inhibit collagen deposition and fibrosis progression (97–99). Autophagy has been proven to inhibit TGF-β1-induced fibrogenesis in human cardiac fibroblasts by targeting TGF-β R II (100). Studies have found that in TGF-β1-activated cardiac fibroblasts, the overexpression of DNA methyltransferase 3A (DNMT3A) inhibits the autophagy of cardiac fibroblasts and promotes their proliferation and fibrosis, characterized by increased expression of α-SMA and collagen I, which affects the progression of CF (101). Heat shock protein HSPA9, a key regulator of fibrosis, is significantly upregulated in atrial fibrillation (AF) and Ang II-induced models. It inversely inhibits autophagic flux through the TGF-β1/Smad pathway, leading to an increase in pro-fibrotic proteins. Knockdown of HSPA9 can relieve this inhibition of autophagy, thereby suppressing the proliferation and migration of fibroblasts and mitigating CF (102).

The PI3K/AKT/mTOR pathway is considered the core regulatory hub connecting energy metabolism, stress response, and autophagic homeostasis. Imbalance of its activity can affect the autophagic process in various ways and ultimately determine the outcome of fibrosis. In the doxorubicin-induced CF rat model, the expression of key proteins in the PI3K/AKT/mTOR pathway was significantly downregulated. Meanwhile, the markers of endoplasmic reticulum stress and autophagy-related proteins were markedly upregulated, suggesting that the excessive activation of autophagy caused by mTOR inhibition might be involved in the fibrotic remodeling process after myocardial injury. In this context, autophagy may no longer serve as only a protective clearance mechanism but may indirectly promote fibroblast activation and collagen deposition (103) by excessively degrading key intracellular structural proteins or inducing cell death. In contrast, in the myocardial injury model induced by chronic intermittent hypoxia, inhibition of the PI3K/AKT/mTOR pathway was also observed. However, the autophagic markers were characterized by a decrease in Beclin-1 and abnormal accumulation of p62, indicating a blockage rather than simple activation of the autophagic flux (104). These studies suggest that the impact of the PI3K/AKT/mTOR pathway on CF does not depend on its activation or inhibition *per se*, but on its regulation of autophagic flux integrity and lysosomal function. The PINK1/Parkin pathway is currently one of the most extensively studied regulatory axes of mitophagy, playing a central role in maintaining mitochondrial quality control and metabolic homeostasis in cardiomyocytes. Notably, PINK1/Parkin-mediated mitophagy can occur in cardiomyocytes themselves. It can also indirectly affect the phenotypic transformation of cardiac fibroblasts by regulating the inflammatory and metabolic signals derived from cardiomyocytes, enhance collagen synthesis and ECM deposition, and promote the progression of CF (105, 106). In a diabetic cardiomyopathy model, high-glucose conditions can impair mitochondrial respiratory function and inhibit the activity of the PINK1/Parkin pathway, accompanied by insufficient mitophagy and energy deficiency. Conversely, activating this pathway can restore mitochondrial function, reduce oxidative stress, and improve cardiac structural remodeling, further verifying its protective role in fibrosis regulation (105). As a mitochondria-targeted hydrogen sulfide donor, AP39 delivers hydrogen sulfide to mitochondria, stabilizes the mitochondrial membrane potential, reduces the production of mtROS, and improves mitochondrial energy metabolism. Given the central role of the PINK1/Parkin pathway in maintaining mitochondrial quality control, AP39 may provide favorable conditions for restoring PINK1/Parkin-dependent mitophagy, thereby indirectly inhibiting the process of CF (107). Therefore, targeting and regulating PINK1/Parkin-mediated mitophagy to restore mitochondrial quality control and energy metabolic balance has become an important strategy for improving CF. In addition, autophagy in immune cells also participates in the regulation of CF. Studies have shown that autophagy in macrophages can be enhanced to reduce the release of pro-inflammatory factors such as IL-1β by inhibiting the activation of inflammasomes, thereby improving the inflammatory state and indirectly alleviating the degree of fibrosis (108). Further research has indicated that a decrease in plasma indole-3-propionic acid (IPA) levels can accelerate aging-related CF. IPA affects the PI3K–AKT–mTOR pathway by regulating PPT1 expression, activates autophagy in senescent macrophages, and inhibits the inflammatory response, thus exerting an anti-fibrotic effect (109). Autophagy plays a complex dual role in cardiac fibrosis, potentially either inhibiting or promoting the development of fibrosis. Future research needs to further explore the specific mechanisms of autophagy under different pathological conditions to develop more precise therapeutic strategies. However, when autophagy is inadequate to restore homeostasis following severe stress, or when apoptotic pathways are suppressed, cells may transition to a form of death that displays a necrotic phenotype and exhibits strong pro-inflammatory characteristics. This programmatic necrosis, which is subject to regulation by specific signaling pathways, is referred to as necroptosis.

### 3.4 Necroptosis

Necroptosis is a typical caspase-independent inflammatory form of programmed cell death mediated by receptor-interacting serine/threonine-protein kinase 1 (RIPK1) and RIPK3 and executed by the downstream molecular effector mixed lineage kinase domain-like protein (MLKL). Morphologically, necroptosis combines the signal regulation characteristics of apoptosis and the membrane rupture characteristics of necrosis, making it an important hub connecting cell death and inflammation (110, 111). During the occurrence and development of cardiac fibrosis, the core pathological significance of necroptosis lies in the death itself and the accompanying release of pro-inflammatory mediators, which can continuously activate cardiac fibroblasts and increase the deposition of ECM. This persistent inflammatory microenvironment not only directly damages surviving cardiomyocytes but also strongly stimulates the activation and proliferation of cardiac fibroblasts. Activated myofibroblasts synthesize and secrete a large amount of ECM components such as collagen, leading to myocardial interstitial fibrosis (112). Therefore, necroptosis is considered a key event connecting cardiomyocyte death, inflammatory amplification reaction, and the initiation of CF.

**Figure 4:**
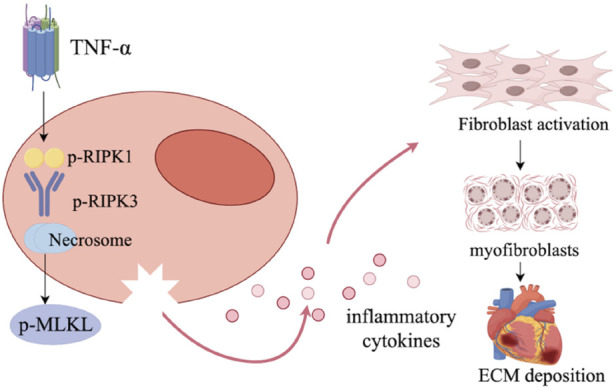
The mechanism of necrotic apoptosis in myocardial fibrosis. Upon stimulation by TNF-α, the necroptotic pathway is activated via phosphorylation of RIPK1 and RIPK3, leading to necrosome formation and downstream activation of p-MLKL. This triggers necrotic membrane rupture and the release of inflammatory cytokines. These cytokines stimulate fibroblast activation, transition into myofibroblasts, and promote ECM deposition, contributing to cardiac fibrosis.

Under pathological conditions such as hypertension, myocardial ischemia/reperfusion, high glucose, or oxidative stress, death receptor ligands like TNF-α bind to TNFR1, promoting the recruitment and phosphorylation of RIPK1. When caspase-8 activity is inhibited, RIPK1 further forms a necrosome with RIPK3 through the RHIM domain, inducing the phosphorylation and oligomerization of MLKL. The activated MLKL translocates to the cell membrane to form pores, leading to the disruption of cell membrane integrity, the leakage of intracellular contents, and a strong inflammatory response, ultimately exacerbating the process of cardiac fibrosis (113, 114) (as shown in Figure 4). In type 2 diabetes models (*in vivo* and *in vitro*) , high glucose and fat induce CF and cardiac dysfunction by activating the RIPK1/RIPK3 pathway and impairing autophagic flux (113). In I/R injury, intervention with the traditional Chinese medicine Arctiin can reduce the levels of RIPK1/p-RIPK1, RIPK3/p-RIPK3, and MLKL/p-MLKL in the hearts of I/R-treated rats and decrease the myocardial infarct size (115). Similarly, Nesfatin-1 has also been shown to alleviate myocardial I/R injury in rats by inhibiting the RIPK1–RIPK3–MLKL axis (116). In a chronic heart failure (CHF) rat model, vascular peroxidase 1 promotes the ubiquitination and degradation of RIPK1 by upregulating the deubiquitinase CYLD, thereby activating the RIPK1/RIPK3/MLKL signaling pathway and exacerbating the programmed necrosis of cardiomyocytes (117). Collectively, these pieces of evidence indicate that the RIPK1/RIPK3/MLKL signaling axis is a key common pathway for cardiomyocyte death and subsequent fibrosis initiation caused by various etiologies. The high etiological non-specificity of this pathway makes it a therapeutic target with great translational potential in CF.

Necroptosis is not merely a membrane rupture event. Its initiation and amplification processes are closely associated with mitochondrial functional imbalance, particularly the activation of the RIPK1/RIPK3 and MLKL pathways, which is closely related to the disruption of mitochondrial homeostasis. During necroptosis, the loss of mitochondrial membrane potential (ΔΨm) and the persistent opening of the mitochondrial permeability transition pore (mPTP) are key factors. In a myocardial injury model induced by pressure overload, stress stimuli can lead to the collapse of mitochondrial membrane potential and the opening of mPTP, further triggering severe disorders in cellular energy metabolism, often manifested as reduced ATP production and decreased mitochondrial DNA copy number. This not only weakens the contractile function of cardiomyocytes but also increases the abnormal activation of cardiac fibroblasts and collagen deposition, thereby promoting the progression of fibrosis (118, 119). Zhang et al. (120) demonstrated that, in addition to MLKL serving as a substrate of RIPK3, calcium/calmodulin-dependent protein kinase II (CaMKII) can also act as a substrate of RIPK3, mediating cardiomyocyte necrosis through the RIPK3-CaMKII mPTP signaling pathway. In I/R injury, after the execution protein MLKL of necroptosis is activated, it can translocate to the mitochondrial membrane to form pores, directly leading to the release of mtROS, exacerbating oxidative stress and generating excessive ROS. This activates downstream inflammatory pathways, promotes the release of inflammatory mediators, further recruits and activates immune cells, stimulates the transformation of cardiac fibroblasts into myofibroblasts, and synthesizes a large amount of ECM proteins, thus promoting the occurrence and development of myocardial interstitial fibrosis (74, 121). Under conditions of I/R and oxidative stress, the expression of synaptotagmin 1 (Syt1) is significantly downregulated, while its overexpression can interact with Parkin to promote Parkin-mediated CypD ubiquitination and inhibit mPTP opening, thereby suppressing cardiomyocyte necroptosis and alleviating interstitial fibrosis (122). Necrostatin-1 (Nec-1), as a classic RIPK1 inhibitor, has shown effects in reducing cell death, reducing infarct size, and improving organ function in models of cerebral infarction, myocardial infarction, and various ischemia–reperfusion injuries. The strategy of combining Nec-1 with the apoptosis inhibitor Z-VAD further indicates that multiple forms of programmed cell death have a synergistic pathogenic effect in myocardial injury (123).

Overall, necroptosis is involved in the occurrence and development of CF through multiple mechanisms such as the inflammatory amplification effect, metabolic–autophagy imbalance, and mitochondrial dysfunction. Targeting the RIPK1/RIPK3/MLKL axis or its upstream regulation of oxidative stress and mitochondrial homeostasis—especially the combined regulation of the necroptotic signaling axis and mitochondrial quality control process—may inhibit the CF process more effectively than single blockade of cell death signals. It is noteworthy that mitochondrial dysfunction and oxidative stress—common hallmarks of necrotic apoptosis—are also key features of ferroptosis, another iron-dependent cell death pathway. Ferroptosis is primarily characterized by lethal lipid peroxidation and the resulting metabolic imbalance.

### 3.5 Ferroptosis

**Figure 5:**
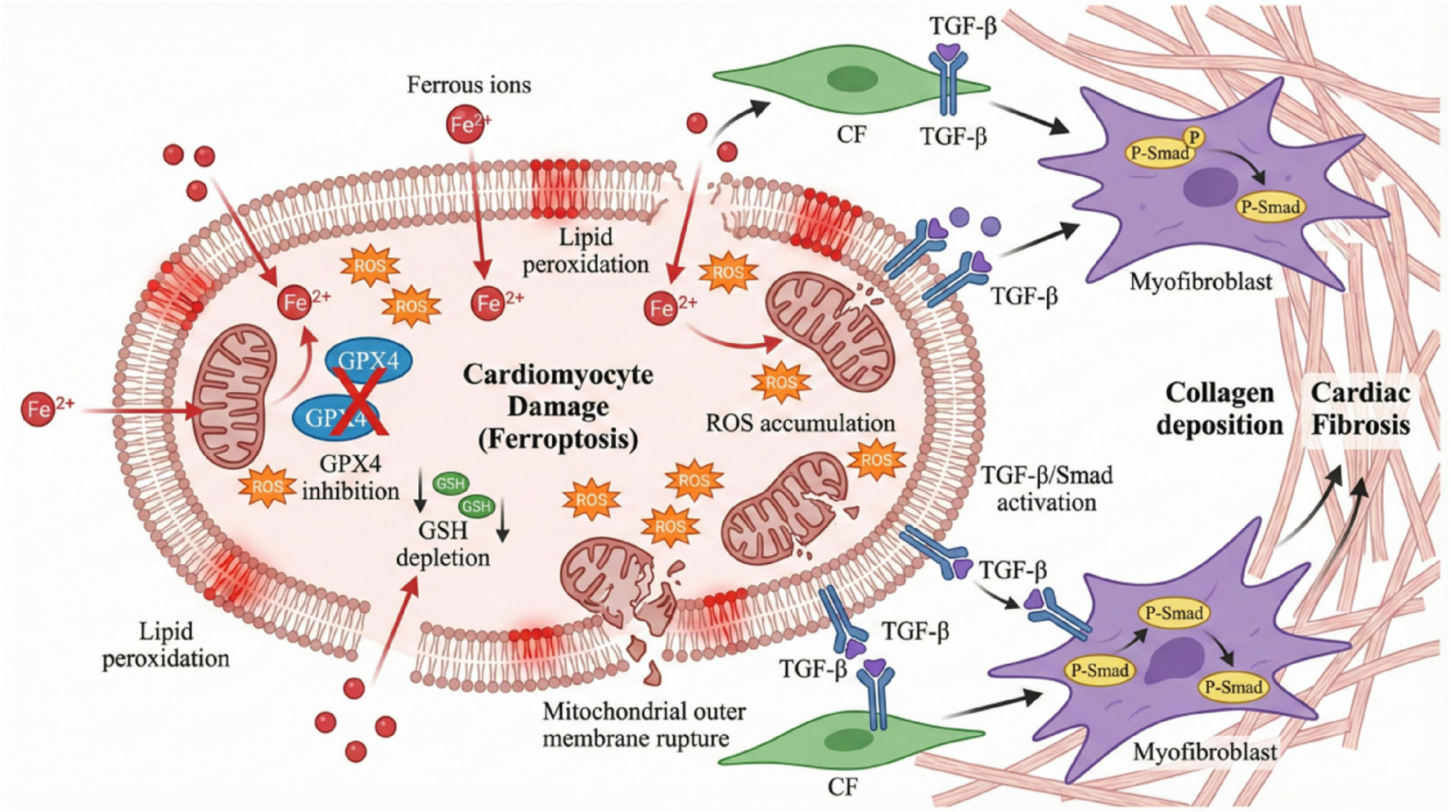
The mechanism of ferroptosis in myocardial fibrosis. In cardiomyocytes, impaired GPX4 activity and GSH depletion lead to lipid peroxidation and membrane damage, characteristic of ferroptosis. Released Fe^2+^ and accumulated ROS further enhance TGF-β signaling, promoting cardiac fibroblast activation and myofibroblast differentiation. This results in fibrotic scar formation and collagen deposition, contributing to cardiac fibrosis.

Ferroptosis is a typical form of iron-dependent programmed cell death, initially induced by the small-molecule compound erastin. Its core features include the accumulation of free Fe^2+^ in cells, abnormal accumulation of lipid peroxides, and imbalance of redox homeostasis (124). Morphologically, ferroptosis is mainly characterized by reduced mitochondrial volume, increased mitochondrial membrane density, and loss of mitochondrial cristae, accompanied by the destruction of the outer mitochondrial membrane integrity, while the nuclear morphology usually remains relatively intact (125). In recent years, studies have shown that ferroptosis plays an important role in the occurrence and development of cardiovascular diseases, particularly cardiac fibrosis. Under pathological conditions, ferroptosis-mediated myocardial cell damage can promote the CF process through multiple mechanisms. After ferroptosis occurs in myocardial cells, lipid peroxidation damage and the release of Fe^2+^ and ROS can directly act on adjacent cardiac fibroblasts, promoting their activation and transformation into myofibroblast phenotypes. The latter excessively synthesizes and secretes collagen, leading to abnormal deposition of ECM and the formation of fibrotic scars (126). In addition, the imbalance of the antioxidant defense system during ferroptosis—particularly the decreased activity of glutathione peroxidase 4 (GPX4) or the depletion of glutathione (GSH)—represents the key molecular basis for uncontrolled lipid peroxidation and the occurrence of ferroptosis. Studies have shown that impaired GPX4 function not only induces ferroptosis in myocardial cells but also activates the TGF-β/Smad signaling pathway, promoting the phosphorylation of Smad proteins, thereby enhancing the trans-differentiation ability of fibroblasts into myofibroblasts and accelerating the progression of CF (127) (as shown in Figure 5). These studies suggest that its core mechanism of ferroptosis primarily revolves around iron metabolism homeostasis and lipid peroxidation, which promote the occurrence of CF. The ferroptosis–fibrosis axis has been verified in various pathological models. In a mouse model of cardiac fibrosis induced by PM2.5, heme degradation in myocardial cells leads to iron overload, which in turn activates the ferroptosis–TGF-β1/Smad3 signaling pathway, an important mechanism driving CF (128). In the ischemia–reperfusion (I/R) injury model, miR-375-3p accelerates ferroptosis in myocardial cells by targeting and inhibiting GPX4, thereby promoting fibrosis. Conversely, the application of miR-375-3p antagomir and the ferroptosis inhibitor Fer-1 can enhance the antioxidant capacity of cardiac fibroblasts and myocardial cells and significantly alleviate I/R-induced CF (129). This study suggests that the MicroRNA–GPX4 axis is an important molecular target for regulating myocardial ferroptosis and its downstream fibrotic response.

Notably, ferroptosis is not an isolated cell death event but has a close cross-regulatory relationship with autophagy, mitochondrial dysfunction, and other forms of programmed cell death. Under conditions of oxidative stress and energy metabolism disorders, iron homeostasis imbalance and mitochondrial damage can jointly amplify the lipid peroxidation reaction, making cardiomyocytes more susceptible to ferroptosis. Meanwhile, abnormal autophagy—especially the selective autophagy process related to ferritin degradation—can further increase the level of intracellular free iron, thus forming a positive feedback loop that promotes the occurrence of ferroptosis. This death network centered on mitochondrial damage–iron metabolism disorder–lipid peroxidation not only exacerbates the loss of cardiomyocytes but also continuously activates cardiac fibroblasts through paracrine and inflammation amplification effects, accelerating the progression of cardiac fibroblasts.

Ferroptosis is associated with the Nrf2/ heme oxygenase-1 (HO-1) antioxidant signaling pathway. Under oxidative stress conditions, ferroptosis can induce the upregulation of HO-1) expression, promote heme degradation, and release free iron, thus forming a positive feedback loop of iron–ROS–lipid peroxidation, which further exacerbates mitochondrial damage and cardiomyocyte death (130). During ferroptosis, damaged cardiomyocytes can also release DAMPs, such as HMGB1, which activate TLR4 and NLRP3 inflammasomes and induce the release of pro-inflammatory factors such as TNF-α, IL-1β, and IL-6. This persistent inflammatory microenvironment not only affects myocardial injury but also directly stimulates fibroblast proliferation and collagen deposition, jointly promoting the occurrence and development of cardiac fibrosis (131). At the molecular regulation level, mixed lineage kinase 3 (MLK3) can mediate oxidative stress and ferroptosis through the regulation of the JNK/p53 signaling pathway, promoting CF in the late stage of chronic heart failure (132). Mitochondrial deacetylase Sirtuin 3 (SIRT3) is considered an important inhibitor of ferroptosis and cardiac fibrosis, and its overexpression can significantly reduce ferroptosis and improve cardiac function (133). In addition, the traditional Chinese medicine compound Fuyu Decoction has been reported to inhibit ferroptosis by activating the Nrf2/GPX4 signaling pathway, thereby improving CF in heart failure (HF) rats (134). In the doxorubicin-induced cardiomyopathy model, excessive lipid peroxidation and mitochondrial-dependent ferroptosis are also considered important pathogenic mechanisms of myocardial injury and fibrosis (135).

In conclusion, ferroptosis is not merely a passive consequence of myocardial injury; instead, it actively participates in and drives the occurrence and progression of cardiac fibrosis by integrating iron metabolism disorders, uncontrolled lipid peroxidation, mitochondrial dysfunction, and amplified inflammatory responses. In this process, ferroptosis of cardiomyocytes not only directly leads to the loss of functional myocardium but also continuously activates cardiac fibroblasts and promotes the maintenance of their pro-fibrotic phenotypes by reshaping the local inflammatory microenvironment and paracrine signaling network. Therefore, ferroptosis can be regarded as a crucial pathological hub connecting myocardial cell injury and abnormal activation of fibroblasts. Intervention strategies targeting key nodes of ferroptosis (such as regulation of iron homeostasis, maintenance of GPX4 activity, activation of Nrf2 signaling, and inhibition of lipid peroxidation) are expected to exert synergistic therapeutic advantages in blocking the CF process. Future research needs to further elucidate the interaction between ferroptosis and other forms of programmed cell death to achieve precise intervention and personalized treatment for CF. However, the landscape of programmed cell death is far from static. Beyond these well-characterized mechanisms, recent studies have unveiled novel, atypical forms of cell death driven by distinct metallic ions and metabolic disturbances, which warrant further attention.

### 3.6 Alternative forms of cell death

#### 3.6.1 Copper-induced cell death

Cuproptosis is a newly proposed form of programmed cell death in recent years. Its core mechanism involves mitochondrial dysfunction and proteotoxic stress triggered by the abnormal accumulation of intracellular copper ions. Studies have shown that excessive copper ions can directly bind to lipoylated proteins in the tricarboxylic acid cycle of mitochondria, promoting protein aggregation and the loss of iron–sulfur cluster proteins, thereby disrupting mitochondrial homeostasis and triggering proteotoxic stress and cell death. In addition, copper ions can also interact with specific copper metabolism-related proteins (such as FDX1 and DLAT) to promote protein oligomerization and mitochondrial structural damage, ultimately leading to cell death (136). In the cardiovascular system, cuproptosis not only directly damages the energy metabolism and mitochondrial function of cardiomyocytes but also participates in the occurrence and development of cardiac fibrosis by activating multiple pro-fibrotic signaling pathways. For example, copper homeostasis imbalance can activate the HIF-1α/TGF-β signaling axis, promoting fibroblast activation and ECM deposition. Meanwhile, the YAP/ATP7A pathway has also been proven to play an important role in the regulation of copper ion-mediated cardiac fibrosis. Research has found that the sodium-glucose cotransporter 2 (SGLT2) inhibitor dapagliflozin can improve cardiac function and alleviate CF, and its mechanism is closely related to reducing copper ion levels in myocardial tissue, decreasing ROS production, and inhibiting the HIF-1α/TGF-β-mediated cell death process (137). In diabetic cardiomyopathy, the high-glucose environment can upregulate the expression of lysyl oxidase-like 2 (LOXL2) by activating the PI3K/Akt/FoxO1 signaling pathway. LOXL2 is a copper-dependent enzyme that can catalyze the cross-linking of collagen molecules, thereby enhancing matrix stiffness and promoting the formation of CF (138). In addition, copper overload can also generate a large amount of ROS through the Fenton reaction, disrupting the mitochondrial oxidative phosphorylation process and leading to energy metabolic disorders in cardiomyocytes (decreased ATP and increased ADP/AMP), further exacerbating myocardial injury and the fibrotic process (139). These findings suggest that cuproptosis and copper metabolism disorders may participate in the regulation of CF through factors such as mitochondrial damage and metabolic disorders.

#### 3.6.2 Parthanatos

Parthanatos is a form of programmed cell death driven by the over-activation of poly ADP-ribose polymerase (PARP), typically associated with DNA damage and oxidative stress. Under conditions of DNA damage, PARP-1 is activated, which can recognize the breakage sites of DNA strands and use nicotinamide adenine dinucleotide (NAD^+^) as a substrate to catalyze the polymerization of ADP-ribose groups to form a large number of poly(ADP-ribose) (PAR) chains. The excessive production of PAR chains can lead to a decrease in intracellular NAD^+^ and ATP and disrupt the mitochondrial membrane potential, prompting the release of apoptosis-inducing factor (AIF) from the mitochondria and its translocation to the nucleus. After AIF enters the nucleus, it can interact with macrophage migration inhibitory factor and other factors to mediate large-scale DNA fragmentation, ultimately resulting in cell death (140).

During the development of CF, pathological factors such as chronic inflammatory response, ischemia–reperfusion injury, and metabolic disorders can lead to the significant production of ROS, triggering persistent oxidative stress. Oxidative stress can directly damage DNA and induce the over-activation of PARP-1, thereby triggering parthanatos. Clinical studies have shown that the level of oxidative stress in patients with chronic heart failure is significantly increased, such as the elevated levels of plasma total peroxides (PRX) and leukocyte lipid peroxidation product 4-hydroxynonenal, accompanied by enhanced activation of PARP-1 and nuclear translocation of AIF, which suggests that parthanatos may be involved in the pathological processes related to myocardial injury and fibrosis (141). In addition, in the I/R injury model, knockout of the ARH3 gene can lead to the abnormal accumulation of PAR mediated by PARP-1, exacerbating tissue damage. The mechanism may be closely related to the over-activation of parthanatos (140). Overall, parthanatos exacerbates the loss of cardiomyocytes through the pathological cascade reaction of DNA damage–PARP-1 over-activation–mitochondrial dysfunction, and may indirectly promote the activation of cardiac fibroblasts through inflammation amplification and paracrine effects, thus playing a potential pro-pathological role in the occurrence and progression of CF. Collectively, these diverse cell death modalities—ranging from classical apoptosis to emerging ferroptosis and cuproptosis—do not operate in isolation within the cardiac microenvironment. Instead, they function as a tightly interwoven network, exhibiting complex crosstalk and mutual regulation.

## 4 Interactions between PCD regulatory networks and myocardial fibrosis

In light of the preceding discussion concerning the diverse modes of cell death, it is acknowledged that these pathways do not function as discrete mechanisms within the intricate microenvironment of myocardial fibrosis. Instead, they form a dynamic and mutually regulated network. Mitochondrial dysfunction, ROS accumulation, and specific signaling hubs—such as RIPK1 and p53—play pivotal roles in this dynamically interlinked system, enabling cells to switch between different death modes based on energy levels and stress intensity. It is imperative to comprehend the mechanisms of crosstalk to elucidate the pathogenesis of myocardial fibrosis and to develop novel therapeutic targets.

### 4.1 Interactions between apoptosis and necroptosis

**Figure 6:**
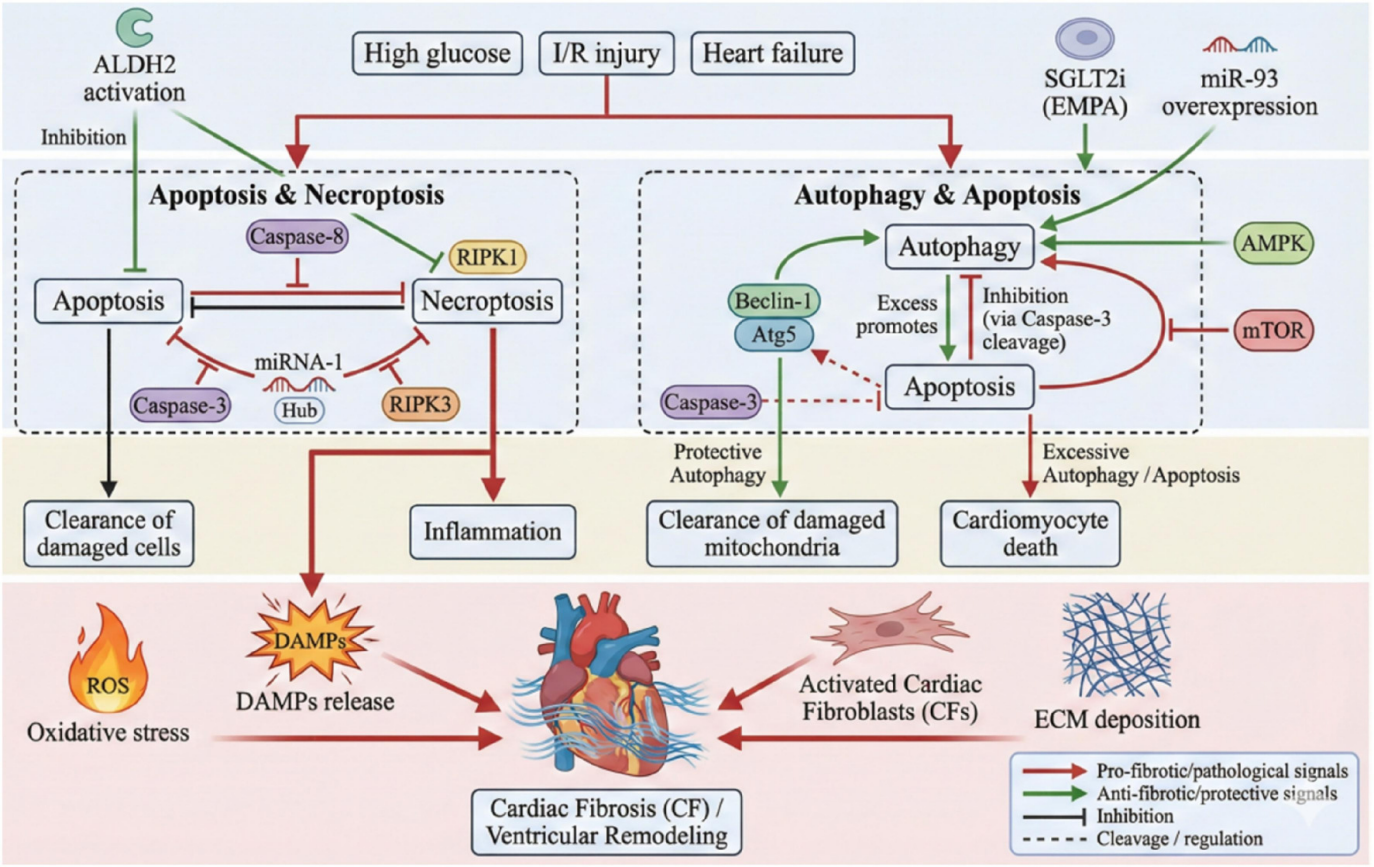
The left module illustrates how apoptosis and necrotic cell death mutually constrain each other via the caspase-8/RIPK1 molecular switch, along with co-regulation by miR-1 and others. Apoptosis relatively limits fibrosis, whereas necrotic cell death exacerbates inflammation by releasing DAMPs and strongly promotes fibrosis. The right module illustrates the bidirectional regulation between autophagy and apoptosis: Protective autophagy suppresses apoptosis, while excessive autophagy or apoptotic feedback can promote cell death. Factors such as EMPA and miR-93 modulate this balance.

Apoptosis and necroptosis are the most common forms of PCD in myocardial tissue, and their roles in myocardial fibrosis are closely related. Generally, apoptosis limits the inflammatory response and the spread of fibrosis to a certain extent by eliminating damaged or abnormal myocardial cells. In contrast, necroptosis amplifies the local inflammatory response and promotes the occurrence of fibrosis due to the destruction of cell membrane integrity, accompanied by the release of a large number of pro-inflammatory factors and DAMPs. At the molecular regulation level, RIPK1 can inhibit the activity of caspase-8, thereby hindering the occurrence of apoptosis. Conversely, caspase-8 can effectively limit the occurrence of necroptosis by cleaving RIPK1 and RIPK3, thus maintaining the balance of cell death modes (142) (as shown in Figure 6). Under certain pathological conditions, the inhibition of apoptosis makes it easier to shift toward the necroptosis pathway, exacerbating the inflammatory response and promoting the development of fibrosis. miRNA-1 affects the activity of caspase-3 by regulating the ubiquitin–proteasome system. The upregulation of its expression not only promotes apoptosis but may also indirectly promote the occurrence of necroptosis by regulating the stability of RIPK3 through the E3 ubiquitin ligase (143). This dual regulation suggests that miRNA-1 may be a hub molecule for the synergistic effect of apoptosis and necroptosis. In a high-glucose-induced myocardial cell injury model, the activation of ALDH2 can inhibit both apoptosis and necroptosis, thereby alleviating myocardial injury and the degree of fibrosis (144). The deficiency of the lipid-metabolic enzyme lipin1 can simultaneously activate caspase-9-dependent apoptosis and RIPK3-dependent necroptosis, leading to sarcolemma damage and calcium overload. This disruption destabilizes myofibrils through the MEF2c transcriptional network. This mechanism may exacerbate fibrosis in myocardial cells through a similar pathway (145). In addition, there may be a competitive relationship between apoptosis and necroptosis. In I/R injury, inhibiting necroptosis may cause the mode of cell death to shift toward apoptosis, and vice versa. This mutual switching between PCD modes not only affects the survival of myocardial cells but also indirectly determines the progression rate and severity of myocardial fibrosis by regulating the intensity of the inflammatory response and paracrine signals (146). In conclusion, the interaction between apoptosis and necroptosis not only expands our understanding of the mechanism of myocardial fibrosis but also provides a theoretical foundation for the development of combination therapies targeting multiple programmed cell death networks.

### 4.2 Interactions between autophagy and apoptosis

Autophagy and apoptosis also exhibit a bidirectional regulatory relationship during myocardial fibrosis. They jointly influence the fibrotic process by regulating cardiomyocyte survival, cardiac fibroblast activation, and ECM remodeling. This interaction relies on multiple signaling pathways, including core pathways such as the Bcl-2 family proteins, the AMPK/mTOR axis, and the caspase cascade reaction.

As the major intracellular degradation pathway, autophagy maintains cardiomyocyte homeostasis by removing damaged organelles and misfolded proteins. In the early stage of myocardial fibrosis, moderately activated autophagy can inhibit apoptosis and protect cardiomyocytes from damage caused by oxidative stress and calcium overload. The mechanism lies in reducing ROS release by removing damaged mitochondria and abnormal proteins, inhibiting the caspase-dependent apoptotic pathway, and preventing the occurrence of apoptosis. However, when autophagic activity is excessively high, it may transform from a protective mechanism into a pro-death signal. It can lead to non-selective degradation of key intracellular structural and functional proteins, trigger energy metabolism disorders, activate the apoptotic signaling pathway, and induce cell death (147–149) (as shown in Figure 6). Conversely, the apoptotic process itself can also regulate autophagic activity in a reverse manner. For example, caspase-3 can cleave autophagy-related protein Beclin-1 and autophagy-related gene (Atg) 5 to generate pro-apoptotic fragments and inhibit autophagosome formation, forming a positive feedback loop that exacerbates cell death (150, 151). Under pathological conditions such as metabolic disorders, the disorder between autophagy and apoptosis becomes more pronounced. In diabetic cardiomyopathy, for example, a high-glucose environment can induce excessive autophagy, leading to non-specific degradation of myocardial structural proteins and cardiomyocyte functional failure. Studies have shown that the SGLT2 inhibitor empagliflozin (EMPA) can regulate the abnormally enhanced autophagic flux back to the physiological range by inhibiting the activity of glycogen synthase kinase-3β (GSK3β). Restoring autophagic homeostasis can not only effectively inhibit cardiomyocyte death but also promote the reactivation of key myocardial transcription factors, thereby reversing diabetes-related myocardial dysfunction and accompanying interstitial fibrotic remodeling (152).

Under physiological or reversible injury conditions, protective autophagy exerts dual anti-apoptotic and anti-fibrotic effects by removing pro-apoptotic stimulating factors. This mechanism is fully demonstrated in the I/R injury model. E1A-like regulator of differentiation 1 (CREG) is considered a key regulatory factor for maintaining the integrity of lysosomal autophagic flux. It promotes the timely removal of damaged mitochondria and misfolded proteins by enhancing lysosomal degradation function, effectively blocking the activation of the mitochondrial-dependent apoptotic pathway (such as cytochrome c release). CREG-mediated enhancement of autophagy reduces cardiomyocyte death and inhibits the alternative fibrotic process triggered by cardiomyocyte death (153).

Notably, autophagy and apoptosis are often coordinately regulated by common upstream signals, providing precise targets for simultaneously intervening in these two forms of programmed cell death. For instance, miR-93 has been identified as an important negative regulator of the RhoA/ROCK signaling pathway. In heart failure models, overexpression of miR-93 inhibits the RhoA/ROCK axis, downregulates the expression of pro-apoptotic molecules such as Bax and caspase-9, promotes the activation of protective autophagy, significantly improves ventricular remodeling, and reduces the degree of myocardial fibrosis (154).

In summary, the interplay between apoptosis, necrotic apoptosis, autophagy, and other forms of programmed cell death reveals the high plasticity of cell fate determination in myocardial fibrosis. This inherent complexity poses a critical therapeutic challenge, as the inhibition of a single pathway may activate compensatory death mechanisms, thereby undermining treatment efficacy. Consequently, the identification and targeting of key molecular nodes within regulatory networks has emerged as a more effective intervention strategy. The theoretical framework thus established serves as the foundational basis for the subsequent discussion of therapeutic strategies.

## 5 Clinical triggers promoting myocardial cell apoptosis and fibrosis

The preceding section elaborated on the intricate molecular network linking programmed cell death (PCD) of cardiomyocytes to cardiac fibroblast activation. However, in clinical practice, these pathological responses are often mediated by specific triggering factors. Therefore, it is imperative to clearly distinguish these triggers to facilitate early risk stratification, prevention, and personalized interventions in cardiac fibrosis. Clinically, cardiomyocyte apoptosis and fibrosis frequently occur concurrently, forming a vicious cycle. The release of signaling molecules by apoptotic cardiomyocytes activates neighboring fibroblasts, thereby promoting fibrosis. Conversely, within the fibrotic microenvironment, deposited extracellular matrix may also induce cardiomyocyte apoptosis through mechanisms such as mechanical stress. The synergistic interaction of these two processes constitutes the core pathophysiological basis for the progression from cardiac compensation to decompensation, ultimately leading to end-stage heart failure. According to the principles of clinical intervenability, the aforementioned factors can be broadly categorized into two distinct categories: non-modifiable and modifiable factors.

### 5.1 Variable factors

Currently, modifiable risk factors serve as core targets for clinical prevention and therapeutic interventions. Through clinical interventions, lifestyle modifications, or pharmacological treatments, the vicious cycle of PCD and fibrosis can be significantly interrupted or delayed. These factors primarily include the following categories.

#### 5.1.1 Myocardial ischemia and reperfusion (I/R) injury

Ischemia/reperfusion (I/R) injury is the most direct modifiable factor clinically inducing programmed cell death (PCD) in cardiomyocytes, commonly observed during revascularization following acute myocardial infarction. Furthermore, it has been identified as a pivotal stimulus for the onset of compensatory (reparative) fibrosis within the myocardium (5). During periods of ischemia, a sudden decrease in ATP production leads to metabolic stress, resulting in the direct activation of intrinsic apoptotic pathways. In the reperfusion phase, the explosive generation of ROS not only damages mitochondrial membranes to induce apoptosis but also triggers lipid peroxidation, leading to ferroptosis (129). Furthermore, calcium overload and the abnormal opening of the mPTP have been identified as the core mechanisms inducing RIPK3-mediated necrotic apoptosis. This abrupt, widespread myocardial cell death releases substantial DAMPs, inducing inflammatory responses that prompt macrophages to secrete TGF-β. This process has been observed to induce the transformation of quiescent fibroblasts into a state of heightened collagen synthesis, resulting in the formation of scar tissue that replaces necrotic myocardium (122). As previously discussed, the multifaceted death networks triggered by I/R injury constitute the initiating phase of pathological cardiac remodeling. The prevailing clinical interventions are chiefly oriented toward decreasing ischemia duration and ameliorating reperfusion injury. Beyond the implementation of early percutaneous coronary intervention to restore blood flow, preclinical studies suggest that the concurrent administration of specific PCD inhibitors (e.g., Ferrostatin-1 for ferroptosis or Nec-1 for necrotic apoptosis) during reperfusion—or the employment of ischemia preconditioning/postconditioning strategies—can effectively suppress ROS bursts and the opening of abnormal mPTP. This approach is designed to maximize the salvaging of dying myocardial tissue at its source, thereby reducing the final extent of fibrotic scar formation.

#### 5.1.2 Mechanical stress and hemodynamic overload

Long-term abnormal hemodynamic loads (e.g., pressure overload induced by hypertension or volume overload caused by valvular regurgitation) are pivotal factors inducing reactive fibrosis (155). As mechanosensitive cells, cardiomyocytes are capable of converting sustained stretch signals into biochemical signals, thereby upregulating pro-apoptotic gene expression via the activation of the MAPK family. Alternatively, they may induce intense ROS activity that activates extrinsic death receptor signaling pathways, directly upregulating pro-apoptotic gene expression (156, 157). Concurrently, persistent pressure overload leads to mitochondrial energy depletion in cardiomyocytes, directly triggering RIPK3-mediated necrotic apoptosis (118). Such loads have been shown to induce reactive fibrosis rather than compensatory repair. Elevated ventricular wall tension has been demonstrated to directly stimulate fibroblast activation, accompanied by abnormal proliferation of local perivascular and interstitial collagen networks, leading to markedly increased myocardial stiffness. For these triggers, the pivotal element of clinical intervention lies in the timely implementation of early unloading. For patients diagnosed with hypertension, achieving and maintaining blood pressure control within the target range recommended by clinical guidelines has been shown to result in the direct elimination of mechanically tension-mediated pro-fibrotic signals. For patients diagnosed with structural valvular disease, the primary treatment strategy entails the timely implementation of surgical procedures such as valve repair or replacement. This approach is predicated on the premise that it can forestall the onset of irreversible, severe, and widespread necrotic apoptosis, as well as diffuse fibrosis, within the myocardium. The rationale underpinning this strategy is to impede mechanically induced cardiac remodeling.

#### 5.1.3 Metabolic dysregulation and glyco-lipid toxicity

Metabolic diseases such as diabetes and obesity are significant systemic triggers that synergistically contribute to the manifestation of multiple PCD patterns. It has been demonstrated that persistent high-sugar and high-fat environments induce glyco-lipid toxicity, leading to abnormal accumulation of advanced glycation end products and toxic intermediates (e.g., ceramides) within cardiomyocytes. This results in a severe impairment of mitochondrial respiratory chain function in cardiomyocytes, leading to excessive accumulation of mitochondrial ROS (158). This oxidative stress state has been demonstrated to initiate intrinsic apoptosis and serve as a core danger signal, activating NLRP3 inflammasomes and inducing caspase-1-dependent pyroptosis (90). Furthermore, glyco-lipotoxicity has been shown to induce endoplasmic reticulum homeostasis imbalance, which is another critical factor linking metabolic disorders to cardiomyocyte death. In models of diabetic cardiomyopathy, the initiation of apoptotic signaling cascades is directly triggered by hyperactivated ER stress, leading to the exacerbation of adverse ventricular remodeling. Conversely, targeted interventions (e.g., modulating the PI3K/AKT/mTOR signaling axis or protein kinase D) that effectively alleviate ER stress significantly suppress cardiomyocyte apoptosis, thereby improving cardiac fibrosis (159). Beyond classical organelle damage, the hyperglycemic environment also upregulates LOXL2 expression via the PI3K/Akt/FoxO1 signaling pathway. This metabolically induced alteration not only triggers copper-mediated cell death in cardiomyocytes but also directly catalyzes irreversible cross-linking of collagen molecules, increasing myocardial biochemical stiffness. Consequently, clinical interventions aimed at addressing metabolic dysregulation must extend beyond the scope of glycemic and lipid control. SGLT2 inhibitors (e.g., empagliflozin, dapagliflozin) have been shown to restore myocardial energy homeostasis and effectively block multiple PCD–fibrosis networks mediated by key factors like HIF-1α/TGF-β.The implementation of rigorous multidimensional metabolic and organelle quality control represents a core intervention strategy to break the vicious cycle of glyco-lipid toxicity, multiple forms of programmed cell death (PCD), and cardiac remodeling.

#### 5.1.4 Abnormal activation of the neurohumoral system

In chronic conditions such as heart failure, the sustained over-activation of the renin-angiotensin-aldosterone system (RAAS) and the sympathetic nervous system transitions from a compensatory mechanism to a cardiotoxic trigger. Ang II, the core effector molecule of the RAAS, exerts its effects by increasing blood pressure via the AT1R receptor. This elevated blood pressure, in turn, exacerbates the mechanical load on the heart. In addition, Ang II directly induces myocardial cells to produce ROS, triggering a cascade of events that lead to apoptosis and necrotic cell death. Sympathetic overstimulation-induced calcium overload has been demonstrated to further exacerbate mitochondrial dysfunction (37). In the context of fibrosis, Ang II and aldosterone have been demonstrated to exert potent direct pro-fibrotic effects by driving fibroblast proliferation and collagen synthesis (183). The most clinically established intervention in current heart failure and cardiac remodeling therapies is the blocking of neurohumoral toxicity. Adherence to guideline-directed pharmacotherapy is paramount, with early combination therapy involving angiotensin-converting enzyme inhibitors/angiotensin receptor blockers (ARBs)/angiotensin receptor–neprilatone (ARNI) (e.g., sacubitril/valsartan), beta-blockers, and aldosterone receptor antagonists (MRA, e.g., spironolactone) being of the utmost importance. This strategy has been shown to competitively block receptors on target organs, thereby interrupting the pathways of oxidative stress, cell death, and fibroblast proliferation that are induced by Ang II and catecholamines.

#### 5.1.5 Environmental factors

Exogenous cardiotoxic substances (e.g., anticancer drugs, environmental toxins, smoking, and alcohol abuse) are well-established chemical stressors that induce myocardial cell death. In this study, we explored the cardiotoxic effects of anthracycline chemotherapeutic agents, such as doxorubicin (DOX), on multiple levels of cell function. These agents have been observed to induce a variety of cell death pathways, including mitochondrial-dependent apoptosis, NLRP3-mediated pyroptosis, and ferroptosis, resulting from excessive lipid peroxidation. Furthermore, exposure to fine particulate matter (PM2.5) in the environment has been shown to specifically activate the ferroptosis-–TGF-β1 pathway (160). Similarly, nicotine in cigarettes has been demonstrated to directly induce apoptosis by activating specific signaling axes (e.g., ERK-4E-BP1) to mediate oxidative stress (161). Consequently, the primary line of defense in anti-fibrotic therapy involves the avoidance of such toxins and the adoption of a healthy lifestyle. The mitigation of these factors necessitates the implementation of preventive interventions and lifestyle modifications. In the case of cancer patients requiring cardiotoxic chemotherapy (e.g., doxorubicin), clinicians have the ability to limit lipid peroxidation and ferroptosis by optimizing cumulative drug doses or prophylactically combining cardioprotective agents (e.g., dexrazoxane). For the general population, the most cost-effective and efficient strategies for eliminating exogenous oxidative stress triggers and implementing primary prevention against myocardial fibrosis are complete smoking cessation, alcohol restriction, and avoidance of environmental toxins like PM2.5.

### 5.2 Immutable factors

Non-modifiable factors are primarily associated with innate genetic background and age. Although they cannot be eliminated through clinical means, they determine an individual's susceptibility to various modifiable stressors, significantly influencing the progression of PCD and fibrosis.

#### 5.2.1 Genetic factors

Genetic background is a pivotal factor in determining susceptibility to cardiac remodeling. On the one hand, genetic polymorphisms or mutations in the genes encoding the key molecules of PCD regulatory pathways—such as the Bcl-2 family, caspases, or ferroptosis-associated GPX4—directly lower the death threshold of cardiomyocytes. This renders individuals susceptible to significant PCD, even in response to mild ischemic or inflammatory stimuli. On the other hand, mutations in genes encoding myosin or nuclear lamellar proteins have been demonstrated to directly induce hereditary cardiomyopathies, such as hypertrophic or dilated cardiomyopathy (162). These structural defects render cardiomyocytes more vulnerable to normal biomechanical stress, readily triggering death programs accompanied by progressive diffuse or replacement fibrosis.

#### 5.2.2 Factors of aging

Aging has been identified as an independent risk factor for programmed cell death (PCD) and fibrosis in cardiomyocytes. With advancing age, basal autophagy capacity within cardiomyocytes declines significantly, accompanied by mitochondrial dysfunction. This phenomenon leads to the natural accumulation of damaged organelles and ROS, resulting in a comprehensive reduction in cardiomyocyte adaptability to stresses such as mechanical load and toxins (163). In addition, aged cardiomyocytes and fibroblasts manifest the SASP, resulting in the spontaneous release of substantial quantities of pro-inflammatory and pro-fibrotic mediators (164). Aging is accompanied by an increase in fibroblast proliferation activity and collagen synthesis capacity that exceeds typical levels. When aging coincides with modifiable risk factors, such as hypertension and metabolic disorders, a synergistic escalation in destructive effects ensues. This precipitates an accelerated onset of myocardial stiffness and heart failure in the elderly population.

**Figure 7:**
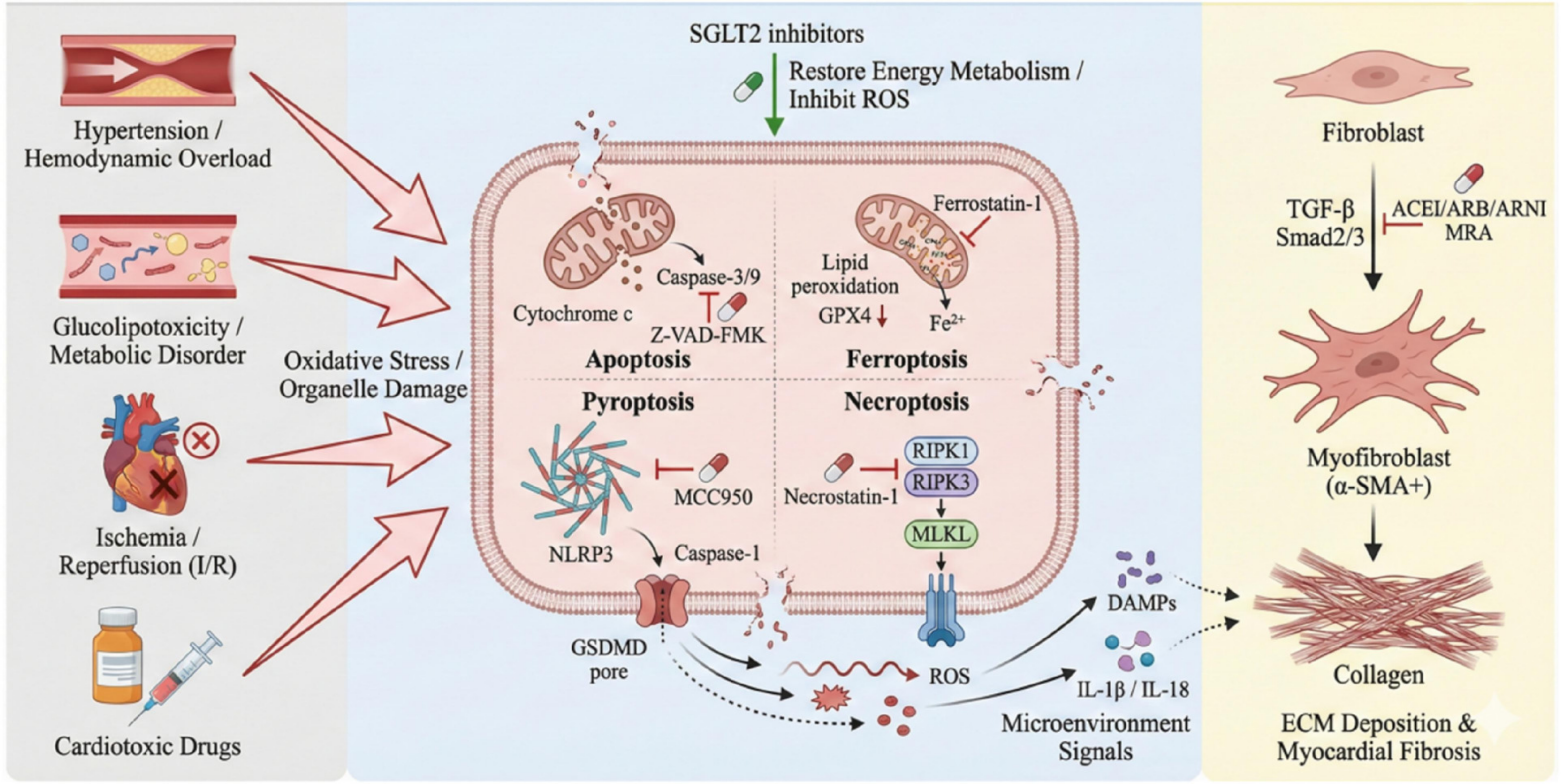
Mechanistic network and potential therapeutic targets for reversing myocardial fibrosis by targeting multiple programmed cell death pathways.

In summary, modifiable and non-modifiable factors interact to collectively regulate the occurrence and progression of multiple forms of PCD and fibrosis in cardiomyocytes. Modifiable factors represent the primary focus for clinical intervention; targeted control can effectively delay or even reverse cardiac pathological remodeling. Concurrently, genetic and age screening for non-modifiable factors facilitates precise assessment of individual disease risk, thereby providing critical guidance for developing personalized anti-fibrotic strategies. As shown in Figure 7, we systematically summarized the pathological axis from macro-level clinical triggers to micro-level multicomponent PCD networks. We comprehensively identified potential therapeutic effect molecules and intervention targets aimed at blocking pathological myocardial remodeling, aiming to provide a panoramic reference for future translational medical research.

## 6 Therapeutic strategies targeting PCD in myocardial fibrosis

### 6.1 Small-molecule inhibitors and agonists

The core pathological features of myocardial fibrosis are the excessive activation of cardiac fibroblasts and the abnormal deposition of ECM. The abnormal regulation of programmed cell death (PCD) is the key mechanism driving this process. In recent years, small-molecule inhibitors or agonists specifically targeting PCD pathways have gradually emerged as potential therapeutic strategies for intervening in myocardial fibrosis. Their mechanisms of action are mainly reflected in reducing the loss of cardiomyocytes, inhibiting the amplification effect of inflammation, and blocking the pathological activation of fibroblasts. The ferroptosis inhibitor Ferrostatin-1 blocks the iron ion-dependent cell death process by scavenging lipid peroxyl radicals, thereby reducing the inflammatory response and the release of fibrotic signals triggered by cardiomyocyte ferroptosis. Cantrell et al. (165) found that Ferrostatin-1 can significantly increase the expression and activity of GPX4 and aconitase, improve the homeostasis of mitochondrial iron–sulfur clusters, and enhance mitochondrial function. These findings suggest that it provides potential therapeutic value for myocardial fibrosis through pathways such as inhibiting ferroptosis, improving mitochondrial function, and reducing oxidative stress (124). In a myocardial infarction model, the intervention of Ferrostatin-1 can reduce the mortality of cardiomyocytes, alleviate the degree of myocardial fibrosis, reduce collagen deposition, and improve cardiac function (165). In addition to directly targeting ferroptosis, the nanozyme strategy based on oxidative stress regulation also provides new ideas for anti-fibrotic treatment. Gu et al. (166) developed a tannic acid (TA)-modified manganese dioxide (MnO_2_) nanozyme. This nanozyme has the dual activities of superoxide dismutase and catalase. It can efficiently scavenge ROS, inhibit the excessive activation of fibroblasts, and further alleviate the process of cardiac fibrosis. Its introduction significantly enhances the affinity of the nanozyme for the cardiac ECM rich in collagen and elastin, improves its retention time and uptake efficiency in myocardial tissue, and demonstrates good anti-fibrotic effects in a mouse model of myocardial infarction. In the therapeutic strategies targeting fibroblasts directly, the precise delivery of anti-fibrotic small molecules also shows unique advantages. In a study on a myocardial infarction model, a delivery system targeting activated cardiac fibroblasts was used to efficiently enrich the sphingosine kinase 1 (SphK1) inhibitor PF-543 in the infarcted area, which significantly inhibited the trans-differentiation of cardiac fibroblasts into myofibroblasts, thereby alleviating the degree of myocardial fibrosis and providing a new strategy for precise anti-fibrotic treatment targeting cardiac fibroblasts (167).

Mitochondrial homeostasis regulation plays a pivotal role in various forms of programmed cell death (PCD) and cardiac fibrosis. *In vivo* and *in vitro* studies indicate that the novel SIRT3 small-molecule activator 2-APQC alleviates isosorbide mononitrate-induced myocardial hypertrophy and fibrosis by improving mitochondrial function and energy metabolism homeostasis, suggesting its potential application as a mitochondria-targeted anti-fibrotic drug (168). With respect to the intervention of the apoptotic pathway, the broad-spectrum caspase inhibitor Z-VAD-FMK has been demonstrated to suppress apoptosis by obstructing the caspase cascade. It has been postulated that this may result in a reduction of reparative fibrosis by decreasing cardiomyocyte apoptosis. Furthermore, under certain conditions, the inhibition of apoptosis may promote a shift toward more inflammatory necrosis, potentially exacerbating fibrosis progression (169). In an adriamycin-induced myocardial injury model, Z-VAD-FMK has been shown to upregulate calumenin expression while suppressing endoplasmic reticulum stress markers GRP78/GRP94 and caspase-3 activation, thereby alleviating ER stress-related cell death and fibrosis (170). Furthermore, it has been demonstrated that the compound may elicit a protective effect by modulating fibrosis-related signaling pathways, such as p38 MAPK (171). In (I/R) injury models, the administration of Z-VAD-FMK has resulted in a substantial reduction in the size of myocardial infarction and an enhancement in cardiac function (172). In the context of inflammatory PCD, the NLRP3 inflammasome inhibitor MCC950 has been shown to possess substantial anti-fibrotic potential. It has been demonstrated that this mechanism functions by impeding the oligomerization and activation of NLRP3, consequently hindering the subsequent activation of caspase-1 and the cleavage of Gasdermin D. This effect culminates in the effective suppression of pyroptosis. MCC950 has been shown to suppress pyroptosis-induced cell membrane rupture, thereby reducing the release of DAMPs, inhibiting GSDMD-N formation, and decreasing the leakage of cellular contents. This effectively inhibits the differentiation of cardiac fibroblasts into myofibroblasts and collagen synthesis, thereby alleviating CF (173).

In summary, small-molecule intervention strategies targeting programmed cell death (PCD) have evolved from the inhibition of a single form of cell death to a new paradigm encompassing multi-pathway synergistic interventions, including ferroptosis, apoptosis, pyroptosis, and mitochondrial function regulation. In the future, the integration of precise PCD regulation, targeted delivery systems, and multi-target combination therapies holds promise for achieving systemic interventions in cardiac fibrosis. These interventions could range from blocking the disease mechanism to facilitating tissue repair.

### 6.2 Gene therapy and epigenetic regulation

#### 6.2.1 CRISPR gene editing

The core of the CRISPR/Cas9 intervention system targeting PCD consists of Cas9 nuclease and single-stranded guide RNA (sgRNA). The sgRNA is composed of a crRNA, which is responsible for sequence-specific recognition, and a tracrRNA, which mediates Cas9 binding. Together, they guide Cas9 to generate double-strand breaks at specific genomic loci, thereby achieving gene knockout, modification, or expression regulation (174). During the occurrence and development of myocardial fibrosis, multiple PCD pathways are abnormally activated and interact with each other, directly or indirectly mediating cardiomyocyte death, inflammation amplification, and continuous activation of fibroblasts. If a certain gene is overexpressed in myocardial fibrosis and promotes the abnormal activation of PCD, CRISPR/Cas9 technology can be used to knock out or modify key genes related to PCD, correcting the PCD abnormality and providing a possibility for the treatment of myocardial fibrosis (175). In the process of myocardial fibrosis, the abnormal activation of necroptosis and pyroptosis exacerbates myocardial injury and fibrosis. RIPK3 and Gasdermin D are closely related to necroptosis and pyroptosis, respectively. Studies have shown that using CRISPR/Cas9 to specifically knock out RIPK3 in cardiac fibroblasts reduces its response to inflammatory stimuli, decreases collagen synthesis, and significantly alleviates myocardial fibrosis (176). In addition, using siRNA delivery systems targeting RIPK3 or Gasdermin D can also inhibit the occurrence of PCD, improving myocardial structural remodeling and the fibrotic phenotype (177). Besides inhibiting pro-death signals, CRISPR technology can also be used to enhance cell survival pathways. In a chronic heart failure model, targeted activation of the Bcl-2 gene, an anti-apoptotic gene in cardiomyocytes, can reduce the apoptosis rate and indirectly inhibit fibroblast activation, thus exerting an anti-fibrotic effect (178). Although CRISPR/Cas9 gene editing shows great potential in the treatment of myocardial fibrosis targeting PCD, its clinical translation still faces multiple challenges, including off-target effects, delivery efficiency, long-term safety, and immune responses. In the future, combining tissue-specific delivery systems and controllable editing strategies is expected to further improve its safety and application prospects.

#### 6.2.2 DNA methylation

DNA methylation, as an important form of epigenetic modification, plays a crucial regulatory role in the occurrence and development of myocardial fibrosis. Abnormal DNA methylation patterns are closely associated with the activation or inhibition of fibrosis-related genes. Hypermethylation in the promoter regions of certain genes can lead to transcriptional silencing, whereas hypomethylation may activate the expression of pro-fibrotic genes (179). Studies have shown that DNA methyltransferases (DNMTs) are the core enzymes for maintaining methylation homeostasis. Among them, DNMT1 and DNMT3b are continuously upregulated in myocardial fibrosis and drive the development of the disease by inhibiting anti-fibrotic genes and activating pro-fibrotic and pro-inflammatory pathways (180). *In vitro* experiments show that hypoxia stimulation can induce a significant pro-fibrotic phenotype in fibroblasts, accompanied by a marked increase in the global DNA hypermethylation level and the expression of DNMT1 and DNMT3b. Blocking the expression of DNMT3b can effectively reduce the levels of type I collagen and α-SMA, thereby inhibiting the abnormal activation of fibroblasts (181). In addition to directly regulating fibrosis-related genes, DNA methylation exacerbates myocardial injury by affecting the PCD pathway. Metes-Kosik et al. found that selenium supplementation could significantly reduce cellular methylation potential, DNMT activity, and overall DNA methylation levels, thereby alleviating CF and improving cardiac systolic function, suggesting a close link between methylation status and cell death regulation (182). Moreover, BMP7 treatment can induce or restore the downregulated expression of TET3 in fibrotic tissues, indicating that BMP7 is upstream of TET3 and initiates subsequent epigenetic modifications to alleviate fibrosis by upregulating the expression or activity of TET3 (183). Studies have shown that in the cardiomyocytes of fibrotic mice, the expressions of DNMT1, caspase-1, and NLRP3 are significantly increased, while the expression of LncRNA-ANRIL is decreased, suggesting a specific regulatory relationship among these genes during the CF process (184).

#### 6.2.3 Histone modifications

Histone modifications—particularly histone acetylation and methylation—serve as crucial epigenetic regulatory mechanisms in the CF process. The determination of cellular fate is achieved through the remodeling of chromatin structure and the modulation of the transcriptional activity of fibrosis-related genes and the PCD pathway. Histone acetyltransferases have been shown to promote chromatin relaxation by increasing histone acetylation levels, thereby activating the expression of pro-fibrotic and pro-inflammatory genes. In contrast, histone deacetylases (HDACs) have been shown to inhibit the transcription of related genes through the process of deacetylation, thereby reducing the expression of pro-fibrotic genes and exhibiting dual regulatory roles in CF (185). Abnormal activation of specific HDAC subtypes has been implicated in the progression of CF across diverse pathological conditions. For instance, in models of diabetic cardiomyopathy, the protein HDAC1 has been shown to promote fibrosis by enhancing histone deacetylation, thereby suppressing the transcription of the anti-fibrotic factor BMP-7. Silencing HDAC1 has been shown to significantly attenuate fibrosis severity, inhibit fibroblast proliferation, and reduce expression levels of collagen I, III, *α*-SMA, and vimentin (186). Furthermore, HDAC6 exerts a non-directive influence on fibrosis-related gene expression and ECM deposition by regulating collagen acetylation status and cytoskeletal remodeling (187). Given the pivotal role of HDACs in fibrosis, HDAC inhibitors have emerged as promising therapeutic strategies. In CHF models, the HDAC inhibitor motegriterat has been shown to possess significant anti-fibrotic properties, with its mechanism of action closely linked to the inhibition of the IL-6/STAT3 signaling pathway. This pharmaceutical agent has been demonstrated to attenuate the pathological upregulation of HDAC1 and HDAC2 in the myocardium of patients with congestive heart failure (CHF). It has been shown to enhance cardiac function, reduce scarring, and decrease collagen deposition, thereby ameliorating adverse ventricular remodeling (188). In addition to HDACs, histone acetyltransferases also play a crucial role in CF. The acetyltransferase p300 is a key enzyme that regulates both histone and non-histone acetylation. In a high-glucose-induced cardiac fibrosis model, p300 enhances TGF-β signaling pathway activity by promoting Smad protein acetylation, thereby driving fibroblast activation and ECM deposition. Its specific inhibitor L002 effectively reverses the hyperglycemia-induced fibrotic phenotype, suggesting that targeting p300 holds potential therapeutic value for anti-fibrosis (189).

Histone methylation is also involved in the regulation of myocardial fibrosis. The histone methyltransferase SET1 promotes pathological myocardial hypertrophy and myocardial fibrosis by mediating the expression of endothelin-1 (ET-1). Knocking down SET1 in endothelial cells can significantly reduce the ET-1 level and alleviate the hypertrophic phenotype of cultured cardiomyocytes (190). In addition, the bromodomain protein BRD4, as a “reader” of acetylated histones, promotes the progression of myocardial fibrosis by recognizing acetylation marks and enhancing the transcriptional activity of pro-fibrotic signaling pathways such as TGF-β. Its inhibitor JQ1 has been proven to significantly improve cardiac function and reduce the degree of fibrosis in various animal models (191). Histone modifications can also form a synergistic network with other forms of epigenetic regulation to jointly affect the PCD and fibrosis process. In fibroblasts of failing hearts, the expression of miR-21 is significantly upregulated. By inhibiting Sprouty1 (Spry1), it enhances the ERK–MAPK signaling activity, thereby regulating the survival, proliferation, and growth factor secretion of fibroblasts, and influencing the degree of myocardial hypertrophy and interstitial fibrosis (24). In addition, the N6-methyladenosine (m6A) modification of RNA—as an important post-transcriptional regulatory mechanism—is also closely related to histone modifications and myocardial fibrosis. In the state of heart failure, the expression of the demethylase FTO is significantly downregulated, leading to an abnormal increase in the overall m6A level in the heart, which in turn affects the stability and translation efficiency of transcripts related to myocardial contractile function and angiogenesis, and ultimately promotes the progression of cardiac dysfunction and fibrosis (192).

Based on these mechanisms, epigenetic therapeutic strategies are advancing from basic research to clinical translation. The epigenetic regulation of myocardial fibrosis involves complex multilevel mechanisms, such as DNA methylation, histone modification, non-coding RNA, and RNA modification. With the in-depth understanding of the epigenetic network, therapeutic strategies targeting specific epigenetic targets are expected to provide new treatment options for myocardial fibrosis. In the future, it is necessary to further strengthen the connection between basic and clinical research to promote the translation of epigenetic therapy from the laboratory to clinical application.

### 6.3 RAAS inhibitors

Sustained over-activation of the renin–angiotensin–aldosterone system (RAAS) is considered one of the core mechanisms driving the occurrence and development of myocardial fibrosis. Its inhibitors can effectively block the pro-fibrotic signaling pathway through multi-target intervention. Ang II—produced by the sequential cleavage of renin or chymase and ACE—is a significant inducer of cardiac fibroblast activation and collagen deposition.

AngII promotes the phenotypic transformation of fibroblasts into myofibroblasts and exacerbates cardiomyocyte apoptosis and inflammatory responses by activating pathways such as TGF-β/Smad, JAK/STAT, and MAPK, thereby amplifying the fibrogenic signals. Both clinical and animal studies have demonstrated that RAAS inhibitors offer significant benefits in improving myocardial structure and functional remodeling. The combined use of mineralocorticoid receptor antagonists (MRAs) and ACE inhibitors can enhance natriuresis, reduce left ventricular filling pressure, and improve ventricular systolic and diastolic function, as well as cardiac output. In a rat model of myocardial infarction, MRA treatment can alleviate adverse ventricular remodeling by promoting the formation of new blood vessels in the infarcted area, manifested as reduced infarct size, thinner left ventricular wall, and decreased degree of dilation (193). Moreover, MRA can delay the progression of myocardial fibrosis by inhibiting the release of pro-inflammatory cytokines, correcting collagen metabolism disorders, and reducing the levels of type III procollagen amino-terminal peptide and matrix metalloproteinases (194). Multiple RAAS inhibitors, including lisinopril, losartan, amlodipine, and spironolactone, have been confirmed to have definite anti-fibrotic effects (53). In patients with hypertension and left ventricular hypertrophy, after 36 consecutive weeks of treatment, compared with the β-blocker atenolol, the angiotensin II receptor antagonist losartan can significantly reduce the myocardial collagen volume fraction (195). In a diabetic cardiomyopathy model, losartan inhibits the expression of TGF-β1 by downregulating the levels of p-JAK2 and p-STAT3 in myocardial tissue, thereby reducing collagen deposition, decreasing left ventricular end-diastolic pressure, and improving cardiac function (196). Moreover, low-dose RAAS inhibitor therapy for patients with diabetes and hypertension can significantly reduce aortic intima-media thickness, myocardial collagen accumulation, and the degree of fibrosis, thus preventing the progression of myocardial fibrosis (197). Furthermore, the anti-fibrotic effect of the aldosterone receptor antagonist spironolactone is not only dependent on the inhibition of Ang II signaling but also has a partially Ang II-independent protective mechanism. In a model of hypertension combined with myocardial infarction, spironolactone can significantly slow down the process of cardiac fibrosis (198). In an experimental autoimmune myocarditis model, it effectively inhibits the development of myocardial fibrosis by suppressing the phosphorylation of Ets-1 and Smad2/3, thereby alleviating the inflammatory response and collagen deposition (199).

Overall, RAAS inhibitors play an important role in modulating key programmed pathological processes—such as myocardial cell apoptosis, inflammatory response, and abnormal activation of fibroblasts at the molecular level—by regulating hemodynamics to improve cardiac load. These combined effects underscore their significant therapeutic value in the RAAS–PCD–myocardial fibrosis regulatory network.

### 6.4 Other inhibitors

In addition to classical signaling pathway inhibitors, in recent years, various drugs with anti-inflammatory, antioxidant, and multi-target regulatory properties have demonstrated good effects in the treatment of myocardial fibrosis. EHP-101 is an oral lipid formulation of the novel non-psychotropic cannabidiol aminoquinone VCE-004. In myocardial tissue, VCE-004 can significantly inhibit the differentiation of cardiac fibroblasts induced by TGF-β and Ang II. Its effects are manifested as a decrease in α-SMA expression, inhibition of ERK 1 + 2 phosphorylation, downregulation of the expression of IL1β, IL6, Col1A2, and CCL2 mRNA in cardiac fibroblasts, and reduction in the transcription levels of pro-inflammatory and pro-fibrotic genes. This consequently reduces collagen deposition in cardiac tissue and exerts a significant anti-fibrotic effect (200). In addition to improving metabolic abnormalities, the glucagon-like peptide-1 receptor agonist liraglutide also exhibits direct cardioprotective effects in hypertensive models. Studies have found that while reducing blood pressure and blood glucose levels, liraglutide can inhibit collagen deposition in cardiac tissue, downregulate the expression of the Ang II type 1 receptor (AT1R), and significantly reduce ROS production, thereby alleviating oxidative stress-related myocardial injury and the fibrotic process (201). Irisin, a myokine discovered in recent years, also shows good anti-fibrotic potential in the Ang II-induced myocardial fibrosis model. Its mechanism mainly relies on activating the Nrf2-mediated antioxidant defense system and simultaneously inhibiting the TGF-β1/Smad3 pro-fibrotic signaling axis, thus significantly reducing the degree of myocardial interstitial fibrosis (202).

**Figure 8:**
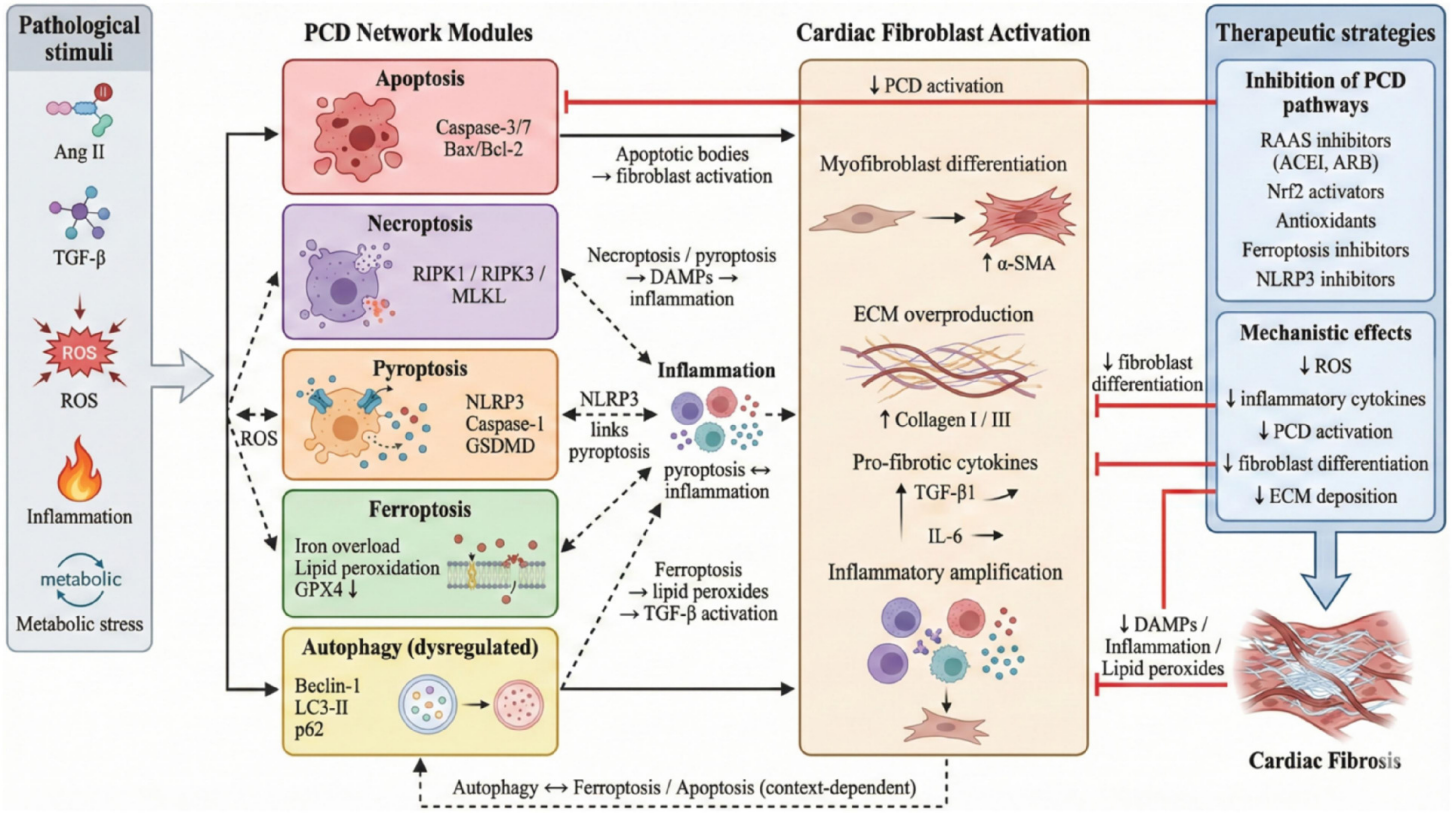
Roles of major programmed cell death (PCD) pathways in cardiac fibrosis and their targeted therapeutic strategies. (Left) PCD types—apoptosis, pyroptosis, necroptosis, ferroptosis, and autophagy—along with their key molecules. These PCD pathways collectively promote fibroblast activation and collagen deposition (middle section) by releasing DAMPs, activating TGF-β signaling, and inducing oxidative stress and inflammatory responses. (Right) Diverse therapeutic strategies targeting these pathways, including small-molecule inhibitors, gene editing, epigenetic modulators, and RAAS inhibitors, offering a multi-targeted therapeutic perspective for CF intervention.

Overall, this type of non-classical inhibitors provides new drug sources and treatment ideas for the intervention of myocardial fibrosis by integrating multiple mechanisms of action, such as anti-inflammation, anti-oxidation, and inhibition of fibroblast activation (as shown in Figure 8). It also lays a foundation for subsequent combined application with targeted strategies for programmed cell death.

## 7 Challenges and future directions

### 7.1 Insufficiencies in mechanism research

Although there is already a certain understanding of the role of PCD in myocardial fibrosis, there are still many deficiencies in mechanism research. There are complex interactions among different types of PCD. During myocardial fibrosis, oxidative stress may simultaneously trigger the signaling pathways related to ferroptosis and pyroptosis, with potential mutual influence and synergistic effects between them. Oxidative stress products generated during ferroptosis may further activate inflammasomes, thereby promoting the occurrence of pyroptosis; conversely, the inflammatory response triggered by pyroptosis may exacerbate oxidative stress and promote ferroptosis. However, the specific molecular mechanisms of this interaction are not yet fully understood, and in-depth research is needed to comprehensively understand the regulatory network of PCD in myocardial fibrosis. In addition, there are differences between animal disease models and human diseases. The physiological characteristics, metabolic patterns of cardiomyocytes, and the regulatory mechanisms of PCD in animals are different from those in human cardiomyocytes. More importantly, their responses to certain drugs and sensitivities to PCD regulation also vary. Therefore, further in-depth research on the mechanism of PCD in human cardiomyocytes is required to improve the clinical relevance of research results.

### 7.2 Barriers in translational medicine

The specificity and safety of drugs targeting programmed cell death (PCD) are significant obstacles in translational medicine. Many drugs targeting PCD may lack sufficient specificity when inhibiting or regulating specific PCD pathways. Although drugs that systemically inhibit apoptosis can reduce myocardial cell apoptosis, they may also inhibit apoptosis in other tissues and cells throughout the body, leading to a series of side effects. Moreover, apoptosis also plays an important role in normal physiological processes such as immune regulation and tissue development. Excessive inhibition of apoptosis may affect these normal physiological functions and trigger adverse reactions. Therefore, the development of specific drugs targeting abnormal PCD pathways in myocardial tissue, while reducing their impact on other tissues and cells, is the key to overcoming this challenge.

### 7.3 Promotion by emerging technologies

Single-cell sequencing technology provides a powerful tool for revealing the cellular heterogeneity of PCD. Traditional sequencing techniques typically analyze a large number of cells and cannot accurately reflect the gene expression characteristics of individual cells. In contrast, single-cell sequencing can sequence the genetic information at the single-cell level, enabling a deeper understanding of the distribution, state, action processes, and cooperation mechanisms of different subpopulations of the same type of cells. In the study of myocardial fibrosis, single-cell sequencing can identify the gene expression differences among different myocardial cell subpopulations during the PCD process and clarify which cell subpopulations are more prone to PCD and their related regulatory mechanisms.

Organoids are three-dimensional cell cultures grown *in vitro* that mimic the structure and function of *in vivo* organs. Organoid technology and artificial intelligence have significant application prospects in drug screening. Cardiac organoids can more realistically simulate the physiological and pathological environments of myocardial tissues and be used to screen anti-myocardial fibrosis drugs targeting PCD. Artificial intelligence can analyze and mine large amounts of biological data to predict the efficacy and safety of drugs, thereby accelerating the drug screening process. For example, by using artificial intelligence algorithms to analyze organoid experimental data, drugs with potential therapeutic effects can be quickly screened out, improving the efficiency of drug development. The continuous development of emerging technologies will bring new breakthroughs in the treatment research of myocardial fibrosis and promote the development of therapeutic strategies targeting PCD.

## 8 Conclusions

Different types of programmed cell death (PCD) make unique contributions to myocardial fibrosis and hold considerable therapeutic potential. Multiple PCD types—including apoptosis, necroptosis, pyroptosis, ferroptosis, and autophagy-related cell death—are involved in the occurrence and development of myocardial fibrosis through their respective signaling pathways and molecular mechanisms. Interventions targeting these different PCD types, such as the use of small-molecule inhibitors and gene therapy strategies, may block or alleviate the process of myocardial fibrosis, providing new targets and ideas for the treatment of myocardial fibrosis. The research on myocardial fibrosis involves multiple fields, including basic research, clinical medicine, and bioengineering. Only through close cooperation and integration of multiple disciplines can we comprehensively understand the pathogenesis of myocardial fibrosis, develop more effective targeted PCD treatment strategies, and ultimately improve treatment outcomes for patients with myocardial fibrosis, enhancing their quality of life and prognosis.
